# Supplementary material for: Positive and relaxed selection associated with flight evolution and loss in insect transcriptomes
Source: Gigascience. 2017 Aug 16;6(10):1–14. doi: 10.1093/gigascience/gix073 (PMC5632299; doi:10.1093/gigascience/gix073)
Supplement: GIGA-D-17-00053_Original-Submission.pdf [file gix073_GIGA-D-17-00053_Original-Submission.pdf]

Manuscript
Click here to download Manuscript Manuscript_Flight_March7,2017.docx

Title:

Positive and relaxed selection associated with flight evolution and loss in insect transcriptomes

Authors:

T. Fatima Mitterboeck[†1,2], Shanlin Liu[†3,4], Sarah J. Adamowicz[1,2], Jinzhong Fu[1], Rui Zhang[3], Wenhui Song[3], Karen Meusemann[5,6,7], Xin Zhou[*8,9]

Affiliations:

[†]shared first authorship

[1] Department of Integrative Biology, University of Guelph, Guelph, ON, Canada N1G 2W1
[2] Biodiversity Institute of Ontario, University of Guelph, Guelph, ON, Canada N1G 2W1
[3] BGI-Shenzhen, Shenzhen, Guangdong Province, China 518083
[4] Centre for GeoGenetics, Natural History Museum of Denmark, University of Copenhagen, Øster Voldgade 5–7, 1350 Copenhagen, Denmark
[5] University of Freiburg, Department for Biology I (Zoology), Evolutionary Biology and Ecology, Hauptstr. 1, D-79115 Freiburg, Germany
[6] Center for Molecular Biodiversity Research, Zoological Research Museum Alexander Koenig, Adenauerallee 160, 53113 Bonn, Germany
[7] Australian National Insect Collection, National Research Collections Australia, Clunies Ross Street, Acton, ACT 2601, Canberra, Australia
[8] Beijing Advanced Innovation Center for Food Nutrition and Human Health, China Agricultural University, Beijing 100193, China
[9] College of Food Science and Nutritional Engineering, China Agricultural University, Beijing 100083, China

***Author for Correspondence:** Xin Zhou, China Agricultural University, Beijing, xinzhoucaddis@icloud.com

Word count: 6123
Table count: 3
Figure count: 3

Additional data deposition: One ‘Additional file’. All nuclear genetic data are available on NCBI (Project PRJNA183205) and some mitochondrial genetic data currently in GenBank (accession numbers provided in Additional file). All data sets, including genetic alignments and scripts, will be deposited into *Giga*DB repository upon acceptance.

ABSTRACT

Background: The evolution of powered flight is a major innovation that has facilitated the success of insects. Previously, studies of birds, bats, and insects have detected molecular signatures of differing selection regimes in energy-related genes associated with flight evolution and/or loss. Here, using DNA sequences from over 1,000 nuclear and mitochondrial protein-coding genes obtained from insect transcriptomes, we conduct a broader test of which gene categories display positive and relaxed selection at the origin of flight as well as with multiple independent losses of flight.

Results: We detected a number of categories of nuclear genes more often under positive selection in the lineage leading to the winged insects (Pterygota), related to catabolic processes such as protease, as well as splicing-related genes. Flight loss was associated with relaxed selection signatures in splicing genes, mirroring the results for flight evolution. Similar to previous studies of flight loss in various animal taxa, we observed consistently higher non-synonymous-to-synonymous substitution ratios in mitochondrial genes of flightless lineages, indicative of relaxed selection in energy-related genes. While oxidative phosphorylation genes were not detected as being under selection with the origin of flight specifically, they were most often detected as being under positive selection in holometabolous insects as compared with other insect lineages.

Conclusion: This study supports some convergence in gene-specific selection pressures associated with flight ability, and the exploratory analysis provided some new insights into gene categories potentially associated with the gain and loss of flight in insects.

Keywords: insect transcriptomes, flight, flight loss, positive selection, 1KITE project, molecular evolution

BACKGROUND

The evolution of active flight in insects has likely positively impacted the species diversity of this group [1]. Flight, having arisen multiple times in animals, arose earliest in insects approximately 406 million years ago and characterizes the clade Pterygota [2]. The evolution of key traits at the origin of Pterygota is not well understood; wings may have originated from the modification of gills, extensions of the body wall, or both [3,4]. By increasing dispersal ability, flight facilitates food and mate finding as well as the avoidance of unfavourable habitats or predators [5]. In addition to the evolution of flight, pterygote insects have at least incomplete metamorphosis, which involves egg, nymph, and adult stages. These transitions paved the way for later innovations, such as wing folding and complete metamorphosis as occurring in holometabolous insects (i.e. egg, larval, pupal, and adult stages), additionally implicated in the evolutionary success of insects [1]. Despite the advantages associated with active flight, flight has been lost an estimated thousands of times in pterygotes [6], such as in lineages representing fleas, snowflies, and stick insects [7].

Powered flight is a highly energetically costly activity in animals, including in birds and bats [8-9]. Flying insects use up to 50 [10] or 100 times [11] more energy when flying than at rest. The oxidative phosphorylation (OXPHOS) pathway in the mitochondrion provides 95% of the energy required for eukaryotic cells [12]. Therefore, the 13 mitochondrial protein-coding OXPHOS genes, the 78 nuclear OXPHOS genes (number present in *Drosophila*) [13], and the 1000+ additional nuclear-encoded genes that function in the mitochondria are likely important in the evolution of traits that require large amounts of energy [9], such as large brain:body size ratios [14]. Genes involved in energy production, such as mitochondrial protein-coding genes, were observed to bear signatures of positive selection with the evolution of flight in animals, or

conversely under relaxed selection with flight loss [8-9,15-16]. However, the association between genes of other functional groups and flight evolution in insects has not been investigated, with previous studies focused on mitochondrial energy-related genes *a priori* [16].

Developmental and gene expression studies have investigated genes relevant to wings or flight ability. Genes important for the physical development of wings have been identified, including the protein-coding genes *wingless, apterous, vestigial, nubbin, nub* [17], and *vein* [18]. Genes differentially expressed in flying and non-flying morphs within certain insect species have also been identified. Genes more highly expressed in flying morphs include 1) those involved in energy production, such as genes that function in the mitochondria [19-20] and the nuclear gene *Isocitrate dehydrogenase* (IDH), which is important in the citric acid cycle [20]; 2) those involved with lipid metabolism [19]; and 3) the *flightin* gene [19-21], which is important for indirect flight muscle function [22]. Genes more highly expressed in flightless morphs include those related to sugar metabolism [19], such as *trehalase* (involved in conversion of trehalose to glucose) [20] and *seryl-tRNA synthetase* (involved in tRNA metabolic processes) [19]. Similar categories of genes could be under differential selection pressures associated with flight gain and loss; however, this has not yet been tested directly with positive selection analysis.

We explore what types of protein-coding genes have experienced differing selective pressures associated with the evolution and loss of flight using DNA sequences from a total of 1476 nuclear single-copy orthologous protein-coding genes and 13 mitochondrial protein-coding genes obtained from transcriptomes. Firstly, we test for evidence of positive selection during the time when flight originated, during a time span of approximately 14 million years [2]. Secondly, we test for positive and relaxed selection among multiple evolutionary losses of flight, which provide more recent and naturally replicated evidence for genes potentially associated with the

evolution and maintenance of flight. In addition to using multiple evolutionary shifts in a biological or ecological trait to identify common genetic trends associated with that shift (e.g. [8,23]), we additionally use the reverse direction event to serve as comparison. Thirdly, to further examine the relationship between energy-related genes and flight, we test for positive selection in available nuclear OXPHOS and mitochondrial OXPHOS genes throughout the major lineages of hexapods.

DATA DESCRIPTION

The nuclear genetic data used in this study consist of transcriptome-derived DNA sequences obtained as part of the 1000 Insect Transcriptome Evolution (1KITE) project (http://www.1kite.org) and additional hexapod genomes, as is presented in Misof et al. [2]. We utilized the current assembly version 2 (strict assembly followed by check for cross contamination, described in [24]) of transcript data of 101 species ([2], NCBI accession PRJNA183205, individual accessions provided in Additional File 1 [AF1] Table S1) and assigned transcripts to 1476 single-copy nuclear orthologous genes included in the ortholog set published by Misof et al. [2]. We additionally included the 12 reference species with an official gene set available and used by Misof et al. [2] to infer orthology; thus data for 113 species were available in total. Orthology assignment of transcripts, alignment, outlier check, alignment refinement, and generation of nucleotide alignments followed the guideline described in Misof et al. [2] with some modifications (see Methods section). Sequences for the 13 mitochondrial protein-coding genes were obtained from the associated mitochondrial transcriptome sequencing project of the Beijing Genomics Institute, with some substitution of sequences from mitochondrial genomes published on NCBI to increase completion (species and sources of data

provided in AF1 Table S13). Mitochondrial sequences were aligned with EMBL-EBI Clustal Omega [25] and Pal2Nal [26]. Guidance [27] was applied to mask sequence regions that were unreliably aligned. The phylogenetic tree topology used here for selection tests was obtained from Misof et al. [2]. The data sets supporting the results of this article will be available in the *Giga*DB repository upon acceptance of this manuscript [reference to be completed upon acceptance].

ANALYSES

Positive selection associated with the origin of flight

Out of 954 nuclear genes tested, 126 (13%) were detected to be under positive selection in the lineage leading to the pterygote insects (‘P’ in Figure 1); 39 of these were uniquely detected to be under positive selection in branch ‘P’ and not detected in either branch ‘U’ (upstream) or ‘D’ (downstream). The 39 unique candidate genes over-represented Gene Ontology categories related to ‘spliceosome’, ‘protein binding’, ‘protease’, and ‘RNA catabolic process’ (Table 1). Nuclear OXPHOS genes were not over- or under-represented in the set of 126 candidate genes under positive selection in lineage ‘P’; only one nuclear OXPHOS gene was in the candidate list out of 13 OXPHOS genes available in the background gene set of 946 genes, which did not differ from the proportional representation expected by chance ($p_{\text{Fisher's exact (2 tailed)}}$=1.0). None of the 13 mitochondrial genes were detected to be under positive selection in the ‘P’ lineage after Benjamini-Hochberg correction.

Table 1. Positively selected genes in the lineage (‘P’) leading to Pterygota as over-represented in A) Gene Ontology (GO) categories from DAVID analysis and B) Biological Process categories

from PANTHER analysis. Terms are for positively selected genes uniquely detected in the ‘P’ lineage and not in two control lineages tested (‘U’ and ‘D’). Here, only categories with $p<0.05$ are shown; full results are given in AF1 Table S11. 954 background genes were mapped to A) 914 IDs and B) 894 IDs; 39 unique candidate genes were mapped to A) 38 IDs and B) 35 IDs.

A) DAVID Gene Ontology results

	914 total genes	38 positively selected genes			
**GO term**	**# in category**	**Expected #**	**Observed #**	**P value**	**Fold enrichment**
precatalytic spliceosome	34	1.4	7	0.00084	5.0
mRNA splicing, via spliceosome	44	1.8	8	0.00087	4.4
catalytic step 2 spliceosome	30	1.2	6	0.0032	4.8
protein binding	81	3.4	8	0.035	2.4
Protease	19	0.8	4	0.038	5.1
mRNA processing	9	0.4	3	0.044	8.0

B) PANTHER Biological Process results

	894 total genes	35 positively selected genes			
**PANTHER GO-Slim Biological Process term**	**# in category**	**Expected #**	**Observed #**	**P value**	**Fold enrichment**
RNA catabolic process (GO:0006401)	9	0.4	2	0.048	5.7

Positive selection associated with flight loss

Eleven lineages (Figure 1) representing flight losses had between 0.8 and 53.7% of genes exhibiting positive selection, with a median of 2.4%. After considering the counts of genes detected under positive selection in the selected related flying lineages, 21 genes were still commonly (in three or more lineages) under positive selection in the flightless lineages. These genes over-represented Gene Ontology categories of ‘coiled coil’ (a protein structural motif), ‘nucleus’, and ‘dendrite morphogenesis’ (Table 2). When considering only the eight fully flightless lineages (excluding female flightless lineages) and seven selected related flying lineages, the Gene Ontology categories for the candidate genes were similar: including the three

listed above, plus ‘DNA binding’, ‘cytosol’, and ‘developmental protein’, and process categories additionally included ‘protein methylation’ (AF1 Table S11).

Table 2. Genes detected to be under positive selection in three or more lineages with flight loss as over-represented in A) Gene Ontology (GO) categories from DAVID analysis and B) Biological Process categories from PANTHER analysis. Counts of positively selected genes in related flying lineages were removed from counts in flightless lineages before functional analysis. In B) child (sub-categorical) processes are indented below parent processes. Only categories with $p<0.05$ are shown; full results are given in AF1 Table S11. 1284 total background genes were mapped to A) 1229 IDs and B) 1207 IDs; 21 candidate genes were mapped to 21 IDs (A and B).

A) DAVID Gene Ontology results

	1229 total genes	21 candidate positively selected genes			
GO Term	# in category	Expected #	Observed #	P value	Fold enrichment
coiled coil	223	3.8	9	0.018	2.4
nucleus	269	4.6	9	0.048	2.0
dendrite morphogenesis	21	0.4	3	0.050	8.4

B) PANTHER Biological Process results

	1207 total genes	21 positively selected genes			
PANTHER GO-Slim Biological Process term	# in category	Expected #	Observed #	P value	Fold enrichment
cellular component organization	113	2.0	5	0.041	2.5
organelle organization	64	1.1	4	0.0229	3.6
chromatin organization	18	0.3	2	0.0387	6.4

Relaxed selection associated with flight loss

Fifty-six out of 1285 nuclear genes tested show significantly higher ($p<0.05$) dN/dS ratios in the flightless pterygote lineages than related flying lineages (red vs. blue lineages in Figure 1). None of these genes overlapped with the 17 genes detected as candidates in the

positive selection analysis of fully flightless lineages. The main GO categories were related to ‘spliceosome’, while processes were ‘RNA localization’, ‘negative regulation of apoptotic processes’, and ‘extracellular transport’ (Table 3). The nuclear OXPHOS genes did not show higher dN/dS ratios in flightless pterygote lineages (note different trees were used per nuclear gene, based on species availability); out of 14 nuclear OXPHOS genes tested, only two had higher dN/dS ratios in flightless lineages with 12 showing higher dN/dS ratios in flying lineages and four of those as significant difference (AF1 Table S9). The myosin binding subunit gene (p=~0) and IDH gene (p=0.050) showed higher dN/dS ratios in flying than flightless lineages. The mitochondrial genes showed significantly higher dN/dS ratios in the flightless pterygote lineages, which here included both-sexes-flightless and female-flightless lineages, than related flying lineages (Figure 2). Eleven out of 13 mitochondrial OXPHOS genes ($p_{binomial} = 0.023$), and all five significant differences, had higher dN/dS ratios in the flightless lineage than in the related flying lineage (p values given in AF1 Table S10).

Table 3. Genes detected to be under relaxed selection (higher dN/dS ratios) in flightless pterygote lineages as compared to related flying lineages as over-represented in A) Gene Ontology categories from DAVID analysis and B) Biological Process categories from PANTHER analysis. In B) child (sub-categorical) processes are indented below parent processes. Only categories with p<0.05 are shown; full results are given in AF1 Table S11. 1285 total background genes were mapped to A) 1231 IDs and B) 1209 IDs; 56 candidate genes were mapped to A) 54 IDs and B) 53 IDs.

A) DAVID Gene Ontology results

GO Term	1231 total genes<br># in category	54 higher dN/dS genes<br>Expected #	 Observed #	P value	Fold enrichment
mRNA splicing, via spliceosome	50	2.2	8	0.0069	3.6
catalytic step 2 spliceosome	35	1.5	6	0.021	3.9
precatalytic spliceosome	39	1.7	6	0.033	3.5

B) PANTHER Biological Process results

PANTHER GO-Slim Biological Process term	1209 total genes<br># in category	53 higher dN/dS genes<br>Expected #	 Observed #	P value	Fold enrichment
RNA localization	11	0.5	3	0.013	6.2
death	13	0.6	3	0.020	5.3
cell death	13	0.6	3	0.020	5.3
apoptotic process	13	0.6	3	0.020	5.3
negative regulation of apoptotic process	1	0.04	1	0.043	22.8
localization	144	6.3	12	0.020	1.9
extracellular transport	1	0.04	1	0.043	22.8

Positive selection in nuclear and mitochondrial OXPHOS genes in hexapod lineages

Six of the 14 nuclear OXPHOS genes present in the total gene set exhibited positive selection in at least one branch (tree with one species chosen per order, represented in Figure 3), along with four of 10 nuclear genes that were randomly selected to use as point of comparison, and three of the five other nuclear genes chosen *a priori* (genes listed in SF Table S12). Each mitochondrial OXPHOS gene had positive selection detected in at least one branch in either the 32-species tree with one species selected per order (Figure 3) or the 66-species tree with multiple species selected per order (results in SF Table S13). The apterygote lineages (grey highlighted) as well as lineages in orders Odonata and Ephemeroptera, which have a direct flight mechanism, did not exhibit many instances of detection of positive selection, excepting in Protura and an interior node (Figure 3). In the mitochondrial tree including more than one species per order,

again no positive selection was detected in apterygotes (excepting in Protura), but some instances of positive selection were detected within the Odonata-Ephemeroptera clade (AF1 Table S13). Occurrence of positive selection in mitochondrial OXPHOS genes was more common in the holometabolous insect clade (labeled 'H' in Figure 3) than in the polyneopteran clade (labeled 'L' in Figure 3); both of those clades contain a similar number of orders and are of similar age (~362 and 387 million years old, respectively [2]). Nuclear genes showed little difference in detection of positive selection between holometabolous and polyneopteran clades (eight vs. seven detections).

DISCUSSION

This study tested for trends in the categories of genes evolving under differing selective pressures associated with flight evolution and loss. The incorporation of both transition directions allows a comparison of trends in the genes under adaptive evolution and relaxed selective constraints with the evolution and loss of flight, respectively. We observed the origin of Pterygota to be associated with detection of positive selection in categories of genes tied to catabolic processes and spliceosome, the latter overlapping with gene categories represented by relaxed selection tests of flight loss. Flight loss was also tied to various categories of genes under positive selection. These tests did not reveal any significant selection pressures in nuclear energy-related genes associated with flight evolution and loss, while mitochondrial genes displayed trends in line with previous expectations of relaxed selection associated with flight loss [8,15-16]. The holometabolous insects had the highest prevalence of signatures of positive selection.

OXPHOS genes related to flight investigated a priori

Energy-related genes, specifically mitochondrial and to a lesser extent nuclear OXPHOS genes, were expected to show signatures of positive selection with the origin of active flight and relaxed selection with the loss of flight. In a study of bat flight evolution [9], the lineage leading to bats was associated with 23% of mitochondrial-encoded OXPHOS genes displaying positive selection, while positive selection was only 3% more common in nuclear OXPHOS genes than in the lineage leading to rodents; other mitochondrial-associated nuclear genes showed no difference between lineages. In our study, no positive selection was observed associated with the origin of Pterygota for the mitochondrial OXPHOS genes, and no over-representation of positive selection was observed for nuclear OXPHOS genes as compared with the background gene sets or with related lineages. It is possible that some signatures of selection were too difficult to detect due to the long time frames, given the trends in mitochondrial and nuclear OXPHOS genes in other insect [15], bird [8], and bat [9] taxa that have evolved or lost flight more recently. The origin of flight in Pterygota occurred approximately 406 million years ago, while bats originated about 60 million years ago [28].

However, associated with flight loss, mitochondrial OXPHOS genes showed evidence of relaxed selection in flightless as compared with flying lineages as demonstrated by significantly higher dN/dS ratios in flightless lineages. This is in accordance with previous observations of proposed relaxed selection in mitochondrial genes associated with flight loss within insect orders [15] and in birds [8]. These findings also mirror molecular patterns in low vs. highly locomotive fish [29] and mammals [8]. Four out of the five significant differences in dN/dS ratios between flightless vs. flying insect lineages were observed in the mitochondrial cytochrome genes (COI, COII, COIII, CytB), while only one significant difference was present for the other

mitochondrial OXPHOS genes. These differences among genes could stem from levels of purifying selection. dN/dS ratios of mitochondrial protein-coding genes in mammals suggest the greatest purifying selection on sequences of COI, COII, COIII, and CytB [30], while in beetles the lowest rates of substitutions at 1st and 2nd codon positions were observed in COI, CytB, ND1, COIII, and COII [31]. Thus, the trends for flightless vs. flying lineage COI, COII, COIII, and CytB genes could be due to greater purifying selection on those genes, in general, thus allowing the effect of relaxed selection to become apparent.

Previously, mitochondrial OXPHOS genes were examined for positive selection throughout insect lineages, and there were fewer signatures of selection detected in apterygote lineages [16]. Here, we included all extant currently described insect orders, improving on the investigation of apterygote hexapod lineages (five orders as compared to two included in Yang et al. [16]) and pterygote lineages (27 orders as compared to 20). As well, we examined nuclear OXPHOS genes. We similarly observed a lack of positive selection in apterygote lineages, and no disproportionate detection of OXPHOS genes evolving under positive selection specifically associated with the origin of Pterygota. Mitochondrial OXPHOS genes exhibited substantial positive selection in the holometabolous insects, while nuclear OXPHOS genes showed little proportional difference in comparison to the control genes. Although the number of taxa included here for holometabolous insects (clade ‘H” in Figure 3) was similar to that for the polyneopteran clade (‘L’), the holometabolous insects represent 83% of all insect species [32]. The detection of selection may in part be linked to the speciation rate of the group, since species diversity and molecular evolutionary rates have been observed to correspond (e.g. [33]). However, this potential mechanism does not fully explain the findings as several highly species-rich groups (such as Lepidoptera) did not exhibit significant positive selection.

It was previously proposed that the type of flight mechanism—asynchronous vs. synchronous flight—may explain trends in adaptive molecular evolution in flying insects [16]. Asynchronous flight, the ability for multiple wing beats per nerve impulse, is present for all of Hymenoptera, Coleoptera, Strepsiptera, Diptera, and Thysanoptera [34]. However, these mechanisms may have similar energetic costs; although synchronous flight may cost more metabolically per stroke, asynchronous fliers often achieve higher stroke frequencies [35-36]. The pattern of positive selection here does not mirror the occurrence of asynchronous vs. synchronous flight. Positive selection associated with the origin of Pterygota was not greater than in downstream lineages. The origin of flight may have set the stage for downstream selection pressures within some lineages related to metabolic efficiency. However, other factors could also be influencing detection of positive selection in particular orders, such as fast mitochondrial gene substitution rates in Strepsiptera [37] ('twisted-wing parasites'), proposed to be due to the transition to parasitism. The trend in holometabolous insects may in general relate to other influences tied to holometaboly itself, such as the occurrence of rapid development, which is thought to constrain genome size in that group [38]. Overall, the pterygotes have a greater occurrence of positive selection in OXPHOS (especially mitochondrial) genes than the apterygotes, as was expected tied to flight ability, with no apparent correspondence to any single flight-related mechanism.

Exploratory analysis of gene categories

In this exploratory analysis, we observed the origin of Pterygota to be associated with signatures of positive selection in protease and RNA catabolic processes genes, whose categories have a common theme of catabolism, which is the subset of metabolic activities involved in

breaking down molecules to release energy and building components. Spliceosome-related genes were also overrepresented in the positive selection results. The origin of Pterygota is associated with other apomorphies other than flight, such as the evolution of metamorphosis and direct sperm transfer, and as such it is possible that results relate to functions other than flight or wings. The fit of Gene Ontology categories with biological expectations would not validate the selection results [39]. However, interestingly, categories ‘proteasome’ and ‘spliceosome’ were also observed to be more highly expressed in flying vs. flightless morphs of aphids [19].

Associated with flight loss, the relaxed gene categories also frequently included ‘splicing’ or ‘spliceosome’. The mirrored occurrence of this category between gain and loss suggests a biological link to flight in insects. While one transcript expression study has linked spliceosome-related genes to flying vs. flightless morphs of cotton aphids [19], citrus and pea aphids do not have this category as a major difference among flying and flightless morphs [20,40], and this category only represents sex-related differences within flying morphs in the brown planthopper [41]. ‘Localization’ was also found to be a general category under relaxed selection in flightless insects, which mirrors the observation of over-representation of expression in localization category between winged vs. unwinged morphs of pea aphids [20].

Alternative splicing of exons in pre-mRNAs is one mechanism that contributes to increased phenotypic complexity [42], and as such, directional selection on splicing mechanisms may be congruent with the evolution of a complex trait such as flight ability. Alternative splicing has a direct necessity to insect flight, which could account for the link with flight loss as well. Almost all structural molecules in insect flight muscles, such as proteins and RNAs, exist as multiple isoforms [43]. Alternative splicing allows various isoforms of muscle-related molecules and as such appears to be an important mechanism to allow for adjustment between energy levels

needed for multiple flight performances [43]. However, it is unclear whether alternative splicing is more frequently occurring for these flight-related genes than all genes in general, as alterative splicing has been observed to occur in a large proportion of genes, at least in humans, including estimates of around 95% of multi-exon genes [44]. In addition, in multiple studies of flying vs. flightless morphs of insects, there are no significant differences in expression levels of splicing genes, suggesting no large difference in general occurrence of splicing in flying vs. flightless insects. However, flight morphs within species have evolved over comparatively short time spans, while here we were looking for differences on macroevolutionary scales. Genes related to splicing are therefore a potential category for further investigation as under differing selection pressures associated with flight and flight loss in insects.

Categories of genes under positive selection associated with flight loss included protein motif (coiled coil), the nucleus, dendrite morphogenesis, and chromatin organization. These do not clearly fit with more highly expressed gene categories in flightless vs. flying morphs of insect species observed by expression studies, such as categories related to sugar metabolism [40] or reproduction (e.g. vitellogenin, an egg yolk protein precursor) [41]. With the loss of flight, and generally reduced dispersal ability, we expected positive selection in genes or processes tied to fecundity, due to the energy trade-off between dispersal and reproduction [20].

Caveats and next steps

The detection of positive selection can be affected by many factors including quality of the sequence alignment [45] and false positives and negatives associated with level of substitution saturation [46]. This study involved detecting positive and relaxed selection along longer timespans than is typical in genome-wide scan studies (e.g. approximately 60 million

years separating dolphin vs. cow [47]). Thus, it is likely that positive or relaxed selection could be difficult to detect due to long time frames and various periods of positive and purifying selection, especially in the lineage leading to Pterygota. While Gene Ontology categories are useful to look for trends in genomic selection, different gene categories could be detected under positive selection with varying species choice, change in background genes available, the Gene Ontology tool [48], or version of tool applied.

The replication provided by multiple losses of flight can help to narrow down uncertainty due to taxon selection and analysis methods, also helping to illuminate the interpretation of the molecular signatures associated with the single evolution of flight. Despite the long timeframes included here, the trends observed for dN/dS ratios in flightless lineages as compared to flying lineages are similar to trends observed on shorter timeframes within insect orders [15] and other animal taxa [8]. Future insect phylogenomic work with increased taxonomic sampling would allow further improvement in the number of cases of flight loss available, with increased accuracy of the phylogenetic mapping of transitions in flight state. Additionally, with larger sample sizes, the effects of co-occurring confounding factors (e.g. parasitism) could be separated, and trends for each various type of flight loss (e.g. female flightlessness vs. full flight loss) could be further investigated.

Importantly, expansion of the loci included in analysis would provide further insight into selection associated with flight gain and loss in insects. The single-copy, transcriptome-derived genes analyzed here represent a portion of all protein-coding genes in the insect genomes and thus restricted the total pool of possible gene categories that could be detected under differing selection pressures; for example, around 16,000 total genes are observed in *Drosophila* species [49]. The orthologous genes included here represent those more essential for life as they are

present and transcribed across a range of arthropod species, life stages, and sexes; many serve basic cellular functions [2]. Thus, genes with more specialized functions, including some related to the development of wings or flying, are likely not represented. Furthermore, there may be important changes in regulatory (non-protein coding) regions, which govern expression levels and the specific tissues in which expression occurs, associated with flight and flight loss. Thus, future comparative genomics analysis using DNA-derived genomes could investigate both protein-coding and non-coding loci, as well as use full genomic data to assess gene gains or losses. Investigation of gene families would likely prove interesting, given that other studies have provided evidence for trends in adaptation based on gene presence and absence or gene family evolution, such as diversification among paralogous genes [49,50].

Conclusions

This study presents an exploratory examination of the genes under positive and relaxed selection associated with the evolution and loss of flight in insects. Considering this study together with prior studies on other animal groups [8-9,15-16], similarities were detected in the selection regime acting upon mitochondrial genes across multiple flying vs. flightless animal groups. These results indicate convergent trends in molecular evolution that parallel convergent functional evolution in evolutionarily disparate animals. Various nuclear gene categories were linked to flight evolution and loss, which could be further explored for potential biological significance. Intriguingly, we found mirror-image patterns of selection in genes relating to splicing: positive selection with the origin of Pterygota and relaxed selection in flightless lineages. The results here contribute insight into the evolution of an important and unique trait that has played a major role in shaping the diversity of life.

METHODS

Genetic data

Generation of the nuclear gene nucleotide alignments from the transcripts included these steps: 1) orthologous transcripts for each species were assigned to 1476 single-copy orthologous genes using an early version of Orthograph [51], version 0.5.4 (available from Github: https://mptrsen.github.io/Orthograph/); 2) each gene was aligned with MAFFT v7.017 [52] using the L-INS-I algorithm for amino acid sequences translated from original nucleotide transcripts during orthology assignment; 3) multiple sequence alignment of each orthologous gene was refined by identification of outlier sequences; refinement of outliers was performed using a profile alignment approach with MAFFT L-INS-I --add; the alignment was again checked for remaining outliers; final removal of outliers was performed; and 4) a modified version (see [2]) of Pal2Nal [26] was applied to obtain the corresponding nucleotide multiple sequence alignments using the protein alignments as blueprint.

Exploratory test of positive selection in lineage leading to Pterygota

Twenty-eight hexapod species were selected to maximize the number of shared nuclear genes available for analysis as well as the phylogenetic representation of pterygotes and non-pterygote hexapods. Not all genes were available for all species in the candidate alignments, and thus species were selected with the trade-off of number of species versus obtaining the largest gene set. We excluded flightless species or orders here from within Pterygota, i.e. representing secondary flight losses. Species selection was performed in a phylogenetically stratified way,

with the final list of 28 species being those that gave the maximum gene count: 1) all five apterygote orders were included, with a maximum of three species per order, but allowing up to one missing sequence per gene for this set; 2) one species from Odonata and one species from Ephemeroptera were included, with no missing sequences allowed; 3) one species per each of five orders of Polyneoptera was included, allowing one missing sequence per gene for this set; 4) one species from each of 10 orders in the clade including Thysanoptera and Diptera (Fig. 1) was included, allowing up to three missing sequences per gene (species selected shown in Fig. 1). This resulted in 954 genes out of 1476. Similarly, 27 species representing apterygote and pterygote hexapod orders were selected for the 13 mitochondrial protein-coding genes, with no missing sequences allowed.

We tested for evidence of positive selection in these nuclear and mitochondrial genes in the lineage leading to Pterygota (Fig. 1 branch 'P'). We used the branch-site method of detecting positive selection [53] in the program PAML *codeml* version 4.8 [54], with the fit of models A1 (non-synonymous-to-synonymous (dN/dS) ratio fixed at 1) vs. A (dN/dS ratio free to vary) (each model with four classes of sites, each class allowing a certain combination of dN/dS ratios representing positive selection, purifying selection, or neutral evolution) compared for each gene separately through likelihood ratio tests [55]. For this and subsequent analyses, we corrected for false discovery due to multiple genes being tested by using the Benjamini-Hochberg correction [56] for each gene within a set, with a family-wise alpha of 0.05.

We repeated the tests on two additional lineages to serve as a null hypothesis to compare to the results for the lineage 'P'. Branch 'U' (upstream) and 'D' (downstream) (Fig. 1) were tested, each representing a time span of an estimated 20 million years [2]. Using these results, we separated out genes that were uniquely detected as being under positive selection in the lineage

leading to pterygote insects. These unique genes were subjected to Gene Ontology (GO) analysis, described in the ‘Functional analysis’ section below.

Exploring genes under positive selection with flight loss

Eleven cases of flight ‘loss’ were identified by mapping flight state on the available phylogenetic tree (Fig. 1) adopted from Misof et al. [2], and three of these cases involved flight loss only in the female sex. Not all of these evolutionary losses were accurately mapped to the correct branch here, given the available species sampled. For example, a loss may have occurred in the common ancestor of a family, but only species representing superfamily-level divergences were available for our analysis. In the case of phasmids, flight loss occurred multiple times within the order [6,57]. However, all available species were flightless, and thus the losses could not be represented accurately on the phylogeny; we tested the branch leading to the phasmid clade to approximate the timing of early flight losses in that order. Due to incomplete phylogenetic mapping of some of the flight loss events, the branches tested here likely represent some flying lineage history in addition to flightless lineage history, which may cause underestimation of molecular signal due to flight loss. A qualitative assessment is provided to indicate the likely degree of accuracy in the mapping of each case of flight loss, considering the density of taxonomic sampling in that group and how frequently flight is thought to have been lost in those groups (Fig. 1 and AF1 Table S4). Sub-trees including the lineage of interest, sister lineage(s), and three successively branching outgroups were used to test for signatures of positive selection associated with each case of flight loss separately in order to maximize gene coverage; no missing gene data were allowed for the species within each sub-tree. Each sub-tree

contained 14 to 19 species, with 584 to 1174 genes available for all species in each analysis (listed in AF1 Table S4). A total of 1284 genes was included, considering all 11 sub-trees.

A test for positive selection was performed on each of the 11 branches of interest for each sub-tree and gene separately. Those genes with significant p values (at 0.05 level after Benjamini-Hochberg correction) within a sub-tree were included in further analysis. We identified genes that were detected as evolving under positive selection in three or more of the 11 lineages tested. However, in order to eliminate those genes exhibiting a signature of selection in many lineages regardless of flight state, we also tested nine flight-capable lineages that were sister lineages or were closely related to the flightless lineages for positive selection using the same sub-trees as the flightless lineages (trees and results in AF1). There were numerous flight loss events in one sub-tree, and so there were fewer related flight-capable lineages to include, resulting in nine flight-capable lineages tested overall (as compared with 11 flightless lineages). The counts of occurrence of positive selection detected ($p<0.05$ after Benjamini-Hochberg correction) were tallied for the flying lineages, and these counts were removed from the list of candidate genes for the flightless lineages. Those remaining genes with three or more counts of positive selection in the flightless lineages were included in functional analysis. This procedure was repeated for the eight cases of full flight loss (i.e. excluding the three cases of female-only flight loss) as compared to seven related flying lineages.

Exploring genes under relaxed selection with flight loss

Nuclear and mitochondrial genes were examined for relaxed selection associated with flight loss using branch models in PAML *codeml* to estimate dN/dS ratios for lineages of interest. For nuclear genes, the total 113-species tree (Fig. 1) was used, and missing data were

allowed. Only genes with data for 80 or more species were included, resulting in 1285 genes tested. Flightless lineages representing full flight loss (not female-only flight loss) were coded one branch rate (red branches in Fig. 1) and the sister or related flight-capable lineages of similar tip number and taxonomic rank were coded together a separate rate (blue branches in Fig. 1), while all other lineages were coded as the background rate.

For each gene, a change in selection regime associated with loss of flight was concluded when there was a significantly increased dN/dS ratio (between 0 and 1) in flightless lineages as compared to flying lineages. Likelihood ratio tests between 3-rate trees (flightless [red], flying [blue], background [black + purple]) and 2-rate trees (flightless [red] + flying [blue] branches vs. all other lineages [black + purple]) were used to test for significant dN/dS differences between target lineages and sister lineages with Chi square test at $p<0.05$. P values were corrected by Benjamini-Hochberg correction across genes. Those genes that had a significantly higher dN/dS ratios in the flightless than flying lineages were examined by functional analysis (below) as compared to the total gene set tested. We interpreted increased dN/dS ratios as signifying relaxed selection. This interpretation of the dN/dS ratios involves the assumption that the majority of non-synonymous changes across a whole gene sequence are selectively neutral or slightly deleterious; by contrast, positive selection is assumed to affect a small minority of sites at which mutations with beneficial effect have occurred [58]. However, given that increased dN/dS ratios can be due to strong positive selection rather than relaxed selection (or in combination, in different parts of the gene), as a precaution we verified whether any genes from this list overlapped with those in the final candidate list for genes under positive selection in both-sexes-flightless lineages.

For mitochondrial genes, a 66-species tree adopted from Misof et al. [2] (similar to Fig. 1) was used, shown in AF1 Table S10. Since there is no 'background' gene set due to all mitochondrial genes being energy related, we directly compared the dN/dS ratios in the flightless vs. related flying lineages. In preliminary tests on these mitochondrial genes and in Mitterboeck and Adamowicz [15], the female-flightless lineages yielded similar results to full-flightless lineages as compared with related flying lineages. Due to this, and the smaller number of flightless lineages in the mitochondrial gene tree, we considered both female- and both-sexes flightless lineages in the flightless category (e.g. red + purple flightless lineages vs. blue flying lineages, with black background lineages).

Functional analysis

We tested for over-representation in Gene Ontology (GO) categories by the genes exhibiting positive or relaxed selection as compared to each total gene set analyzed ('background genes') using the DAVID (Database for Annotation, Visualization and Integrated Discovery) version 6.8 (October 2016) Functional Annotation chart tool [59,60] to identify enriched annotation terms and similarly using PANTHER (Protein Analysis Through Evolutionary Relationships) version 11.1 [61] to identify 'processes'. The genes were matched to *Drosophila* genome functional annotations where available, using the FlyBase ID (http://flybase.org/) for each gene. No additional false discovery rate correction was applied (p values are raw) as correction was already applied for the positive selection analysis, and also the number of candidate genes was lower than the optimal working input for DAVID (hundreds to thousands of genes [59]). For DAVID analysis the expected number of genes in each GO category was calculated by the number of genes in the category divided by the number of background genes,

multiplied by the number of candidate genes (e.g. expected number in Table 1 first GO term; (34/914)*38 = 1.4). The fold enrichment was calculated by the observed number divided by the expected number of genes in that GO term. The 1476 source genes used in our analysis are not representative of gene categories in the full insect genomes; we provide information on the gene categories over- or under-represented by these 1476 genes in relation to the full genome of *Drosophila melanogaster* in AF1 Table S14. Our tests for over-representation of genes under positive or relaxed selection are in relation to each of our available gene sets, i.e. 'background' sets that are each a subset of the total 1476 genes.

Positive selection in energy-related genes in Hexapoda

Specific genes were investigated that related to energy production or were *a priori* hypothesized to be related to flying or flight loss. These included 14 nuclear OXPHOS genes available in the total gene set (1476 genes) identified via their FlyBase IDs, which are a subset of the 78 OXPHOS genes listed in Tripoli et al. [13], and five other genes of interest identified by name or description in DAVID functional annotation: *wingless*, *IDH*, *flightless1*, *myosin binding subunit*, and an energy-related gene (Dmel_CG1271). Ten additional genes with full species coverage were pseudo-randomly selected (not considering function, with the selections spread out by FlyBase IDs) and also analyzed to check for phylogenetic biases in the positive selection results. We selected one species per hexapod order (32 orders) and one species from each of two arthropod outgroups (outgroups were available for the nuclear genes only; 34 species total), for each set of nuclear and mitochondrial genes. In selecting species, we considered gene completeness, with preference for those species available across the most genes of interest. In a few cases, substitutions of some species were made to improve gene completeness (species lists

provided in AF1 Table S12). Mitochondrial genes, where gene sampling was more complete for species, were additionally tested with more than one species per order (up to 6 species) to investigate effects of species sampling on the results; the 66-species tree was the same as that used for relaxed selection analysis of mitochondrial genes (in AF1 Table S10). Tests for positive selection were conducted on all lineages using the program HyPhy [62] and the Branch-site REL (Random Effects Likelihood) model [63] implemented on the publically-available DataMonkey server [64].

ADDITIONAL FILE

Additional file 1. Contains input and output information such as gene lists, newick trees, p values for selection tests, and functional analysis results (MS Excel 2.5MB)

Abbreviations

GO: Gene Ontology; PAML: Phylogenetic Analysis by Maximum Likelihood; AF1: Additional File 1

Acknowledgements

We thank Lili Zhou for her contribution during the early stages of this project. We give a huge thank you to the 1KITE community, who have made the data possible, especially Alexander Donath and Lars Podsiadlowski, who worked on the current transcriptome assembly, cross contamination check, and submission to NCBI. We thank Stephen Marshall and Daniel Ashlock for input on the ideas in earlier versions of this manuscript.

Funding

This work was supported by the University of Guelph (Integrative Biology PhD Award, Dean's Tri-council Scholarship, and Ontario Graduate Fellowship to T.F.M.), the Government of Ontario (Ontario Graduate Fellowship to T.F.M.), and by the Natural Sciences and Engineering Research Council of Canada (Alexander Graham Bell Canada Graduate Scholarship to T.F.M., Discovery Grants 386591-2010 to S.J.A. and 400479 to J.F.).

Availability of data and materials

The datasets supporting the results of this article will be available in the *Giga*DB repository associated with this publication [reference to be completed upon acceptance]. The nuclear sequencing data are available associated with the NCBI Project PRJNA183205.

Authors' contributions

Conceived or designed work: X.Z., T.F.M., R.Z., W.S., J.F., S.J.A., S.L. Filtered genetic data: K.M., S.L. Designed data sets and analyses: T.F.M., S.L. Conducted bioinformatics for PAML analyses: S.L. Conducted Gene Ontology and HyPhy analyses: T.F.M. Drafted the article, generated figures and tables: T.F.M. Revised article drafts: T.F.M., S.J.A., S.L., J.F., K.M. All authors have read and approved the final manuscript.

Competing interests

The authors declare that they have no competing interests.

REFERENCES

1. Mayhew PJ. Why are there so many insect species? Perspectives from fossils and phylogenies. Biol Rev. 2007;82:425–54.

2. Misof B, Liu S, Meusemann K, Peters RS, Donath A, Mayer C, et al. Phylogenomics resolves the timing and pattern of insect evolution. Science. 2014;346:763-767.

3. Averof M, Cohen SM. Evolutionary origin of insect wings from ancestral gills. Nature. 1997;385:627–30.

4. Clark-Hachtel CM, Linz DM, Tomoyasu Y. Insights into insect wing origin provided by functional analysis of vestigial in the red flour beetle, *Tribolium castaneum*. Proc Natl Acad Sci USA. 2013;110:16951–6.

5. Grimaldi D, Engel MS. Evolution of the Insects. New York: Cambridge University Press; 2005.

6. Whiting MF, Bradler S, Maxwell T. Loss and recovery of wings in stick insects. Nature. 2003;421:264–7.

7. Roff DA. The evolution of flightlessness in insects. Ecol Monogr. 1990;60:389–421.

8. Shen Y-Y, Shi P, Sun Y-B, Zhang Y-P. Relaxation of selective constraints on avian mitochondrial DNA following the degeneration of flight ability. Genome Res. 2009;19:1760–5.

9. Shen Y-Y, Liang L, Zhu Z-H, Zhou W-P, Irwin DM, Zhang Y-P. Adaptive evolution of energy metabolism genes and the origin of flight in bats. Proc Natl Acad Sci. 2010;107:8666–71.

10. Roff A. Life history consequences of bioenergetic and biomechanical constraints on migration. Am Zool. 1991;31:205–15.

11. Krogh A, Weis-Fogh T. The respiratory exchange of the desert locust (*Schistocerca gregaria*) before, during and after flight. J Exp Biol. 1951;28:344–57.

12. Erecinska M, Wilson DF. Regulation of cellular energy metabolism. J Membr Biol. 1982;70:1–14.

13. Tripoli G, D’Elia D, Barsanti P, Caggese C. Comparison of the oxidative phosphorylation (OXPHOS) nuclear genes in the genomes of *Drosophila melanogaster*, *Drosophila pseudoobscura* and *Anopheles gambiae*. Genome Biol. 2005;6:R11.

14. Ai W-M, Chen S-B, Chen X, Shen X-J, Shen Y-Y. Parallel evolution of IDH2 gene in cetaceans, primates and bats. FEBS Lett. 2014;588:450–4.

15. Mitterboeck TF, Adamowicz SJ. Flight loss linked to faster molecular evolution in insects. Proc R Soc B-Biological Sci. 2013;280:20131128.

16. Yang Y, Xu S, Xu J, Guo Y, Yang G. Adaptive evolution of mitochondrial energy metabolism genes associated with increased energy demand in flying insects. PLoS One. 2014;9:e99120.

17. Brook WJ, Diaz-Benjumea FJ, Cohen SM. Organizing spatial pattern in limb development. Annu Rev Cell Dev Biol. 1996;12:161–80.

18. Paul L, Wang S-H, Manivannan SN, Bonanno L, Lewis S, Austin CL, et al. Dpp-induced Egfr signaling triggers postembryonic wing development in *Drosophila*. Proc Natl Acad Sci USA. 2013;110:5058–63.

19. Yang X, Liu X, Xu X, Li Z, Li Y, Song D, et al. Gene expression profiling in winged and wingless cotton aphids, *Aphis gossypii* (Hemiptera: Aphididae). Int J Biol Sci. 2014;10:257–67.

20. Brisson JA, Davis GK, Stern DL. Common genome-wide patterns of transcript accumulation underlying the wing polyphenism and polymorphism in the pea aphid (*Acyrthosiphon pisum*). Evol Dev. 2007;9:338–46.

21. Xue J, Zhang XQ, Xu HJ, Fan HW, Huang HJ, Ma XF, et al. Molecular characterization of the flightin gene in the wing-dimorphic planthopper, *Nilaparvata lugens*, and its evolution in Pancrustacea. Insect Biochem. Mol Biol. 2013;43:433–43.

22. Vigoreaux JO, Hernandez C, Moore J, Ayer G, Maughan D. A genetic deficiency that spans the flightin gene of *Drosophila melanogaster* affects the ultrastructure and function of the flight muscles. J Exp Biol. 1998;201:2033–44.

23. Foote AD, Liu Y, Thomas GWC, Vinař T, Alföldi J, Deng J, et al. Convergent evolution of the genomes of marine mammals. Nat Genet. 2015;47:272–5.

24. Mayer C, Sann M, Donath A, Meixner M, Podsiadlowski L, Peters RS, et al. BaitFisher: a software package for multispecies target DNA enrichment probe design. Mol Biol Evol. 2016;33:1875–86.

25. Sievers F, Wilm A, Dineen D, Gibson TJ, Karplus K, Li W, et al. Fast, scalable generation of high-quality protein multiple sequence alignments using Clustal Omega. Mol Syst Biol. 2011;7:539.

26. Suyama M, Torrents D, Bork P. PAL2NAL: Robust conversion of protein sequence alignments into the corresponding codon alignments. Nucleic Acids Res. 2006;34:609–12.

27. Penn O, Privman E, Ashkenazy H, Landan G, Graur D, Pupko T. GUIDANCE: A web server for assessing alignment confidence scores. Nucleic Acids Res. 2010;38:23–8.

28. Meredith RW, Janec JE, Gatesy J, Ryder OA, Fisher CA, Teeling EC, et al. Impacts of the cretaceous terrestrial revolution and KPg extinction on mammal diversification. Science. 2011;334:521–4.

29. Strohm JHT, Gwiazdowski RA, Hanner R. Fast fish face fewer mitochondrial mutations: patterns of dN/dS across fish mitogenomes. Gene. 2015;572:27–34.

30. Castellana S, Vicario S, Saccone C. Evolutionary patterns of the mitochondrial genome in Metazoa: exploring the role of mutation and selection in mitochondrial protein–coding genes. Genome Biol Evol. 2011;3:1067–79.

31. Pons J, Ribera I, Bertranpetit J, Balke M. Molecular phylogenetics and evolution nucleotide substitution rates for the full set of mitochondrial protein-coding genes in Coleoptera. Mol Phylogenet Evol. 2010;56:796–807.

32. Foottit RJ, Adler PH. Insect Biodiversity: Science and Society. Chichester, UK: John Wiley & Sons; 2009.

33. Eo SH, Dewoody JA. Evolutionary rates of mitochondrial genomes correspond to diversification rates and to contemporary species richness in birds and reptiles. Proc R Soc B Biol Sci. 2010;277:3587–92.

34. Resh VH, Carde RT, editors. Encyclopedia of Insects, 2nd Edition. Elsevier; 2009.

35. Evans PD, Wigglesworth VB. Advances in insect physiology. Florida, USA.: Academic Press Inc.; 1988.

36. Conley KE, Lindstedt SL. Energy-saving mechanisms in muscle: the minimization strategy. J Exp Biol. 2002;205:2175–81.

37. McMahon DP, Hayward A, Kathirithamby J. The first molecular phylogeny of Strepsiptera (Insecta) reveals an early burst of molecular evolution correlated with the transition to endoparasitism. PLoS One. 2011;6:e21206.

38. Gregory TR. Genome size and developmental complexity. Genetica. 2002;115:131–46.

39. Pavlidis P, Jensen JD, Stephan W, Stamatakis A. A critical assessment of storytelling: Gene ontology categories and the importance of validating genomic scans. Mol Biol Evol. 2012;29:3237–48.

40. Shang F, Ding B, Xiong Y, Dou W, Wei D, Jiang H, et al. Differential expression of genes in the alate and apterous morphs of the brown citrus aphid, *Toxoptera citricida*. Nat Sci Reports. 2016;6:32099.

41. Xue J, Bao Y-Y, Li B-L, Cheng Y-B, Peng Z-Y, Liu H, et al. Transcriptome analysis of the brown planthopper *Nilaparvata lugens*. PLoS One. 2010;5:e14233.

42. Keren H, Lev-Maor G, Ast G. Alternative splicing and evolution: diversification, exon definition and function. Nat Rev Genet. 2010;11:345–55.

43. Marden JH. Functional and ecological effects of isoform variation in insect flight muscle. In: Vigoreaux JO, editor. Nature's Versatile Engine Insect Flight Muscle Inside and Out. New York: Springer Science+Business Media; 2006. p. 214–29.

44. Pan Q, Shai O, Lee LJ, Frey BJ, Blencowe BJ. Deep surveying of alternative splicing complexity in the human transcriptome by high-throughput sequencing. Nat Genet. 2009;40:1413–6.

45. Mallick S, Gnerre S, Muller P, Reich D. The difficulty of avoiding false positives in genome scans for natural selection. Genome Res. 2009;19:922–33.

46. Gharib WH, Robinson-Rechavi M. The branch-site test of positive selection is surprisingly robust but lacks power under synonymous substitution saturation and variation in GC. Mol Biol Evol. 2013;30:1675–86.

47. Sun Y-B, Zhou W-P, Liu E-Q, Irwin DM, Shen Y-Y, Zhang Y-P. Genome-wide scans for candidate genes involved in the aquatic adaptation of dolphins. Genome Biol Evol. 2012;5:130–9.

48. Faria D, Schlicker A, Pesquita C, Bastos H, Ferreira AEN, Albrecht M, et al. Mining GO annotations for improving annotation consistency. PLoS One. 2012;7:e40519.

49. Hahn MW, Han M V, Han S-G. Gene family evolution across 12 *Drosophila* genomes. PLoS Genet. 2007;3:e197.

50. De Grassi A, Lanave C, Saccone C. Genome duplication and gene-family evolution: the case of three OXPHOS gene families. Gene. 2008;421:1–6.

51. Petersen M, Meusemann K, Donath A, Dowling D, Liu S, Peters SR, et al. Orthograph: a versatile tool for mapping coding nucleotide sequences to clusters of orthologous genes. BMC Bioinformatics. 2017;18:111.

52. Katoh K, Standley DM. MAFFT multiple sequence alignment software version 7: improvements in performance and usability. Mol Biol Evol. 2013;30:772–80.

53. Zhang J, Nielsen R, Yang Z. Evaluation of an improved branch-site likelihood method for detecting positive selection at the molecular level. Mol Biol Evol. 2005;22:2472–9.

54. Yang Z. PAML 4: phylogenetic analysis by maximum likelihood. Mol Biol Evol. 2007;24:1586–91.

55. Yang Z. Likelihood ratio tests for detecting positive selection and application to primate lysozyme evolution. Mol Biol Evol. 1998;15:568–73.

56. Benjamini Y, Hochberg Y. Controlling the false discovery rate: a practical and powerful approach to multiple testing. R Stat Soc Ser B. 1995;57:289–300.

57. Stone G, French V. Evolution: Have wings come, gone and come again? Curr Biol. 2003;13:R436–8.

58. Hughes AL. Looking for Darwin in all the wrong places: the misguided quest for positive selection at the nucleotide sequence level. Heredity. 2007;99:364–73.

59. Huang DW, Sherman BT, Lempicki RA. Bioinformatics enrichment tools: paths toward the comprehensive functional analysis of large gene lists. Nucleic Acids Res. 2009;37:1–13.

60. Huang DW, Sherman BT, Lempicki RA. Systematic and integrative analysis of large gene lists using DAVID bioinformatics resources. Nat Protoc. 2009;4:44–57.

61. Mi H, Poudel S, Muruganujan A, Casagrande JT, Thomas PD. PANTHER version 10: expanded protein families and functions, and analysis tools. Nucleic Acids Res. 2016;44:D336-42.

62. Pond SLK, Frost SDW, Muse SV. HyPhy: hypothesis testing using phylogenies. Bioinformatics. 2005;21:676–9.

63. Pond SLK, Murrell B, Fourment M, Frost SDW, Delport W, Scheffler K. A random effects branch-site model for detecting episodic diversifying selection. Mol Biol Evol. 2011;24:1–13.

64. Delport W, Poon AFY, Frost SDW, Kosakovsky Pond SL. Datamonkey 2010: A suite of phylogenetic analysis tools for evolutionary biology. Bioinformatics. 2010;26:2455–7.

FIGURE LEGENDS

Figure 1. Tree topology and species used in analyses of nuclear genes. Species names followed by a star indicate those species used in positive selection analysis associated with the origin of Pterygota (branch 'P') and other lineages for comparison (branches 'U' and 'D'). Circles or squares on the branches indicate each of the 11 lineages that were used in positive selection analysis of flight loss, with circles indicating full flight loss and squares indicating female-only flight loss. Triangles indicate related flight-capable branches used for comparison with the flight-loss lineages in positive selection analysis. Note that sub-trees were used for the positive selection tests and so not all species shown here were included. Shading of circles or squares indicate the estimated degree of accuracy that the flight loss was represented by the available lineages (green = good, orange = fair, grey = approximate). Red lineages (full flightless) were compared with blue lineages (related flying) in the nuclear gene analyses of relaxed selection (dN/dS ratios) associated with flightlessness, with all other lineages used for a background rate. A similar (smaller) tree was used for mitochondrial gene analyses of relaxed selection where both red (full flightless) and purple (female-only flightless) lineages were compared with blue (related flying) lineages, with other lineages representing the background rate.

Figure 2. dN/dS ratios in flightless vs. related flying lineages for 13 mitochondrial protein-coding genes. In 11 of 13 genes, the dN/dS ratio in the flightless pterygote lineages is higher than the dN/dS ratio of flying lineages. Genes with significant difference in rates (after Bonferroni-Hochberg correction) are given with '*'; in all five cases, the dN/dS ratio is higher in the flightless lineages than in their flight-capable counterparts. Dashed lines signify the mean dN/dS

values; flightless: 0.031, and flying: 0.021. The tree with lineages tested is provided in AF1 Table S10.

Figure 3. Positive selection in hexapod lineages in nuclear and mitochondrial genes of interest. The tree is adopted from Misof et al. [2], showing orders, and involving one species representative per insect order for each gene tested. Orders/lineages that are shaded grey are apterygote (primarily flightless) and those shaded red have all species flightless due to a loss of flight; note Embioptera and Strepsiptera are female flightless only. The lineage marked with ‘P’ represents the lineage leading to the clade Pterygota; ‘L’ = polyneoptera, ‘H’ = holometabola (complete metamorphosis) insects.

Figure1

Click here to download Figure Figure1_png.png

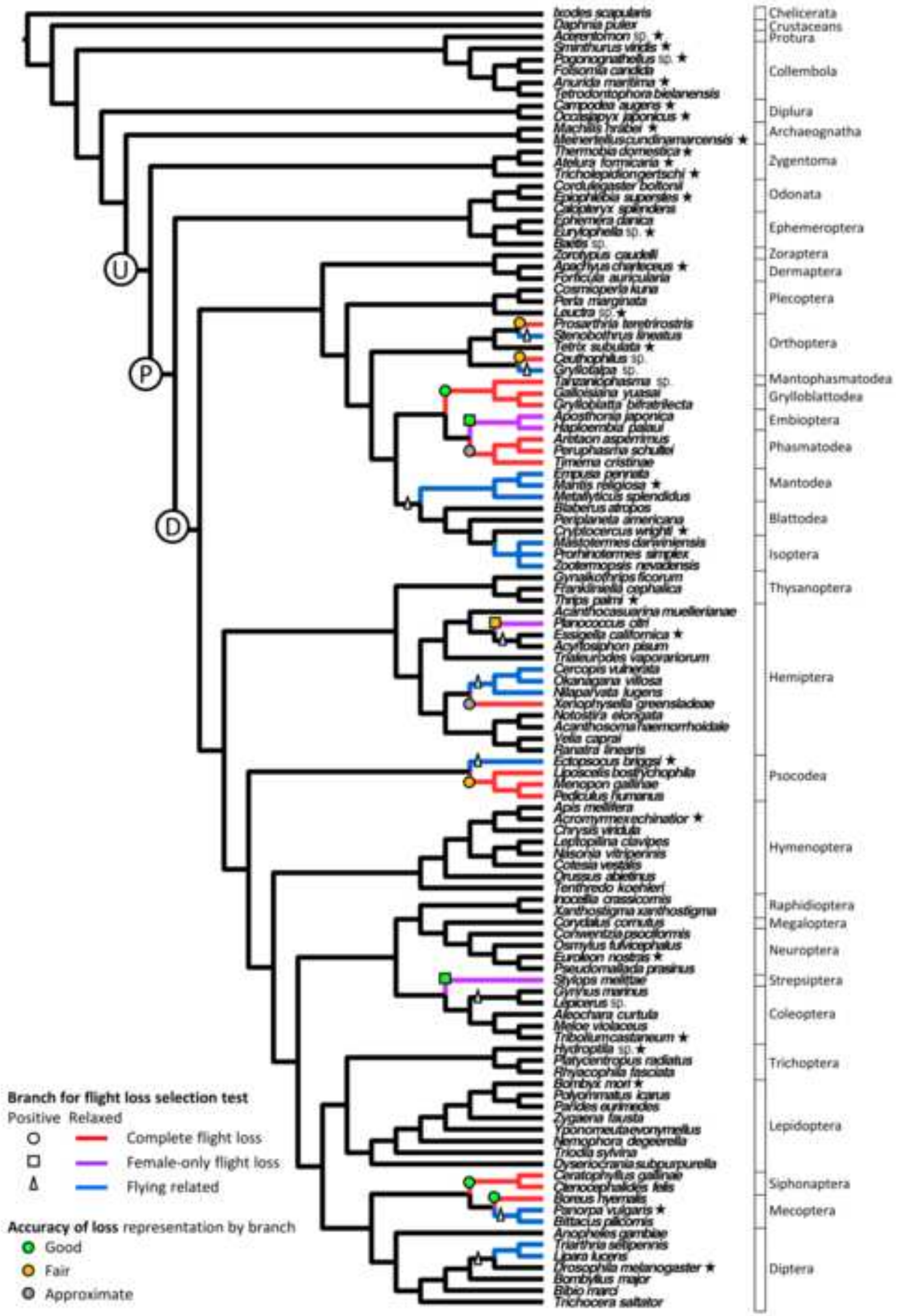

Click here to download Figure Figure2_png.png

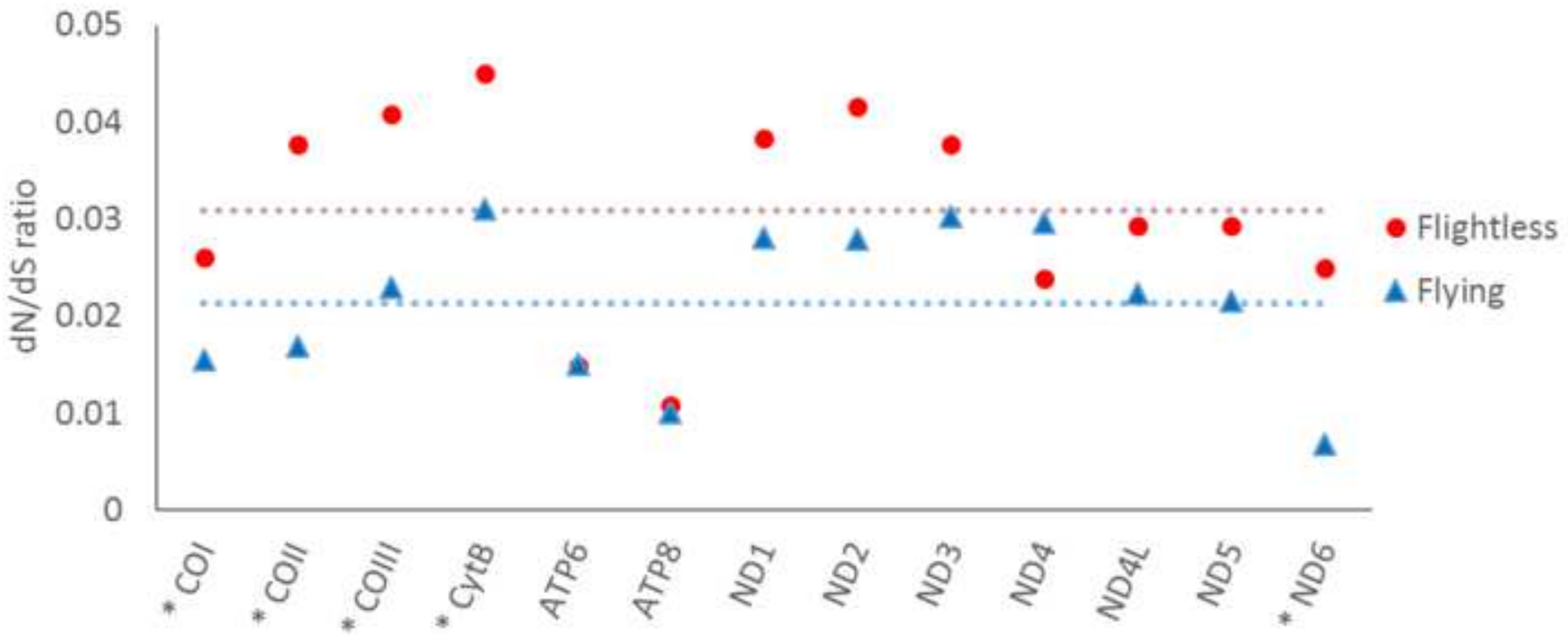

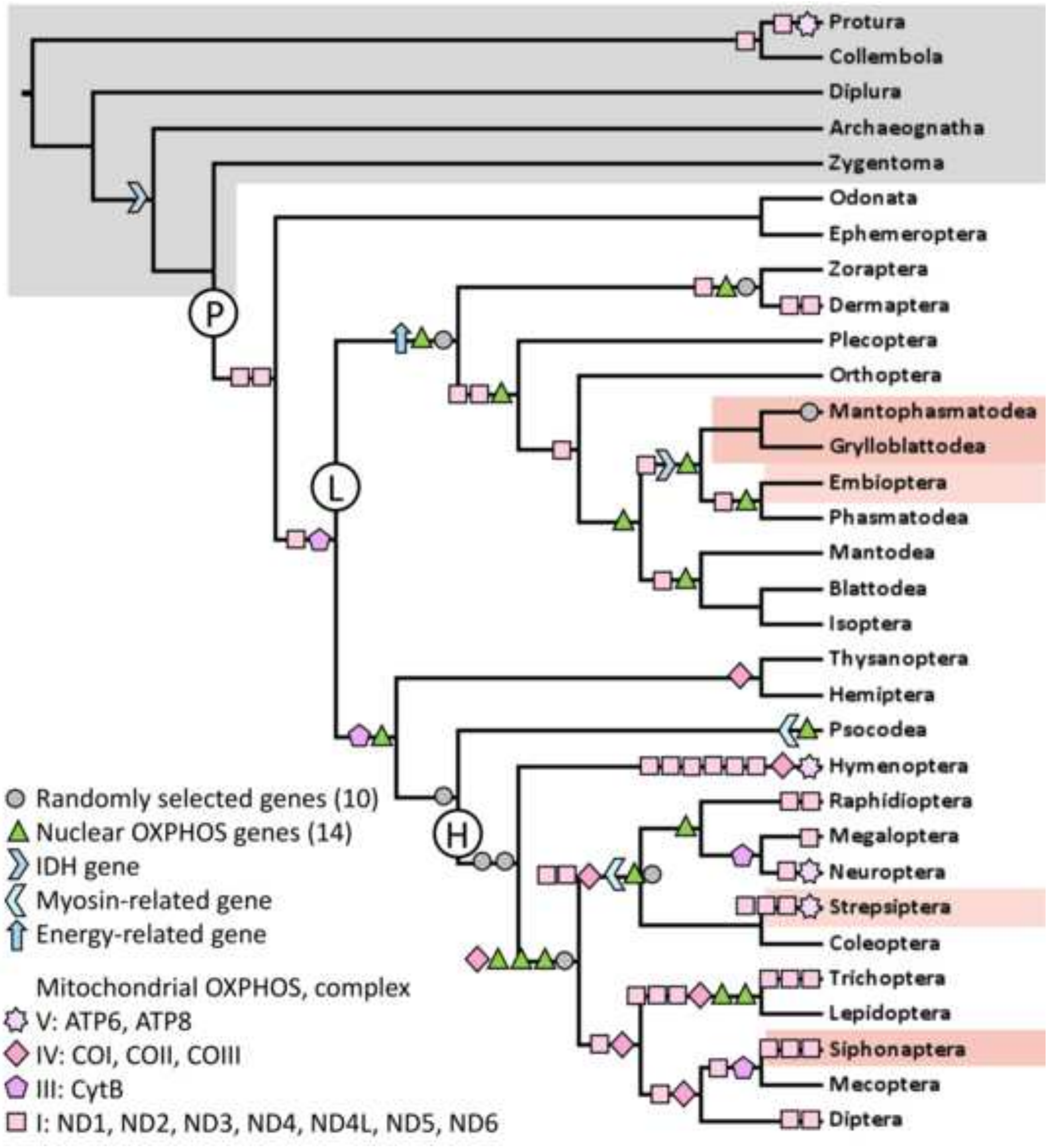

Protura
Collembola
Diplura
Archaeognatha
Zygentoma
Odonata
Ephemeroptera
Zoraptera
Dermaptera
Plecoptera
Orthoptera
Mantophasmatodea
Grylloblattodea
Embioptera
Phasmatodea
Mantodea
Blattodea
Isoptera
Thysanoptera
Hemiptera
Psocodea
Hymenoptera
Raphidioptera
Megaloptera
Neuroptera
Strepsiptera
Coleoptera
Trichoptera
Lepidoptera
Siphonaptera
Mecoptera
Diptera
P
L
H
Randomly selected genes (10)
Nuclear OXPHOS genes (14)
IDH gene
Myosin-related gene
Energy-related gene
Mitochondrial OXPHOS, complex
V: ATP6, ATP8
IV: COI, COII, COIII
III: CytB
I: ND1, ND2, ND3, ND4, ND4L, ND5, ND6

Additional File 1

Click here to access/download
Supplementary Material
AdditionalFile1_(MS Excel).xlsx
