# Supplementary material for: Positive and relaxed selection associated with flight evolution and loss in insect transcriptomes
Source: Gigascience. 2017 Aug 16;6(10):1–14. doi: 10.1093/gigascience/gix073 (PMC5632299; doi:10.1093/gigascience/gix073)
Supplement: GIGA-D-17-00053_Revision-1.pdf [file gix073_GIGA-D-17-00053_Revision-1.pdf]

Manuscript Click here to download Manuscript Mitterboeck_July14,2017_ed.docx

Positive and relaxed selection associated with flight evolution and loss in insect transcriptomes

Authors:

T. Fatima Mitterboeck[†1,2], Shanlin Liu[†3,4], Sarah J. Adamowicz[1,2], Jinzhong Fu[1], Rui Zhang[3], Wenhui Song[3], Karen Meusemann[5,6,7], Xin Zhou[*8,9]

Affiliations:

[†]shared first authorship

[1] Department of Integrative Biology, University of Guelph, Guelph, ON, Canada N1G 2W1
[2] Biodiversity Institute of Ontario, University of Guelph, Guelph, ON, Canada N1G 2W1
[3] BGI-Shenzhen, Shenzhen, Guangdong Province, China 518083
[4] Centre for GeoGenetics, Natural History Museum of Denmark, University of Copenhagen, Øster Voldgade 5–7, 1350 Copenhagen, Denmark
[5] University of Freiburg, Department for Biology I (Zoology), Evolutionary Biology and Ecology, Hauptstr. 1, D-79115 Freiburg, Germany
[6] Center for Molecular Biodiversity Research, Zoological Research Museum Alexander Koenig, Adenauerallee 160, 53113 Bonn, Germany
[7] Australian National Insect Collection, National Research Collections Australia, Acton, ACT 2601, Canberra, Australia
[8] Beijing Advanced Innovation Center for Food Nutrition and Human Health, China Agricultural University, Beijing 100193, China
[9] College of Plant Protection, China Agricultural University, Beijing 100193, China

*__Author for Correspondence:__ Xin Zhou, China Agricultural University, Beijing, xinzhou@cau.edu.cn

Table count: 3
Figure count: 3

ABSTRACT

Background: The evolution of powered flight is a major innovation that has facilitated the success of insects. Previously, studies of birds, bats, and insects have detected molecular signatures of differing selection regimes in energy-related genes associated with flight evolution and/or loss. Here, using DNA sequences from over 1,000 nuclear and mitochondrial protein-coding genes obtained from insect transcriptomes, we conduct a broader exploration of which gene categories display positive and relaxed selection at the origin of flight as well as with multiple independent losses of flight.

Results: We detected a number of categories of nuclear genes more often under positive selection in the lineage leading to the winged insects (Pterygota), related to catabolic processes such as proteases, as well as splicing-related genes. Flight loss was associated with relaxed selection signatures in splicing genes, mirroring the results for flight evolution. Similar to previous studies of flight loss in various animal taxa, we observed consistently higher non-synonymous-to-synonymous substitution ratios in mitochondrial genes of flightless lineages, indicative of relaxed selection in energy-related genes. While oxidative phosphorylation genes were not detected as being under selection with the origin of flight specifically, they were most often detected as being under positive selection in holometabolous (complete metamorphosis) insects as compared with other insect lineages.

Conclusion: This study supports some convergence in gene-specific selection pressures associated with flight ability, and the exploratory analysis provided some new insights into gene categories potentially associated with the gain and loss of flight in insects.

Keywords: insect transcriptomes, flight, flight loss, positive selection, 1KITE project, molecular evolution

BACKGROUND

The evolution of active flight in insects has most likely had a positive impact on the species diversity of this group [1]. Flight, having arisen multiple times in animals, arose earliest in insects approximately 400 million years ago and characterizes the clade Pterygota [2]. The evolution of key traits at the origin of Pterygota is not well understood; wings may have originated from the modification of gills, extensions of the body wall, or both [3][4][5]. By increasing dispersal ability, flight facilitates the finding of food and mates as well as the avoidance of unfavourable habitats or predators [6]. In addition to the evolution of flight, pterygote insects evolved incomplete metamorphosis, which involves egg, nymph, and adult stages. These transitions paved the way for later innovations within Pterygota, such as wing folding and complete metamorphosis as occurring in holometabolous insects (i.e. egg, larval, pupal, and adult stages), which are additionally implicated in the evolutionary success of insects [1]. Despite the advantages associated with active flight, it has been estimated that flight has been lost thousands of times within pterygotes [7], such as in lineages representing fleas, snowflies, and stick insects [8].

Powered flight is a highly energetically costly activity in animals, including in birds and bats [9][10]. Flying insects use up to 50 [11] or 100 times [12] more energy when flying than at rest. The oxidative phosphorylation (OXPHOS) pathway in the mitochondrion provides 95% of the energy required for eukaryotic cells [13]. Therefore, the 13 mitochondrial protein-coding OXPHOS genes, the 78 nuclear OXPHOS genes (number present in *Drosophila*) [14], and the hundreds of additional nuclear-encoded genes that function in the mitochondria (postulated in *Drosophila melanogaster*) [15] are likely important in the evolution of traits that require large amounts of energy [10], such as large brain:body size ratios [16]. Genes involved in energy

production, such as mitochondrial protein-coding genes, were observed to bear signatures of positive selection with the evolution of flight in animals, or conversely under relaxed selection with flight loss [9][10][17][18]. However, the association between genes of other functional groups and flight evolution in insects has not been investigated, with most previous studies focused on mitochondrial energy-related genes *a priori* [18].

Developmental and gene expression studies have investigated genes relevant to wings or flight ability. Genes important for the physical development of wings have been identified, including the protein-coding genes *wingless, apterous, vestigial, nubbin, nub* [19], and *vein* [20]. Genes differentially expressed in flying and non-flying morphs within certain insect species have also been identified. Genes more highly expressed in flying morphs include 1) those involved in energy production, such as genes that function in the mitochondria [21][22] and the nuclear gene *Isocitrate dehydrogenase* (IDH), which is important in the citric acid cycle [22]; 2) those involved with lipid metabolism [21]; and 3) the *flightin* gene [21][22][23], which is important for indirect flight muscle function [24]. Genes more highly expressed in flightless morphs include those related to sugar metabolism [21], such as *trehalase* (involved in conversion of trehalose to glucose) [22] and *seryl-tRNA synthetase* (involved in tRNA metabolic processes) [21]. Functions of genes observed to be differentially expressed between flying insect individuals with higher vs. lower flight metabolic rate include ribosome/RNA processing [25], while genes exhibiting differences between long- vs. short-distance flight migrators include those involved in lipid mobilization and flight muscle structure [26]. Additionally, particular splice forms of certain genes such as encoding glycerol-3-phosphate dehydrogenase (functions in the glycolytic pathway to produce ATP) appear necessary for flight [27], with the relative abundance of various splice variants affecting the power output of flight muscles, as shown in a dragonfly species [28].

Similar categories of genes could be under differential selection pressures associated with the evolutionary gain and loss of flight; however, this has not yet been tested directly with selection analysis.

We explore what types of protein-coding genes have experienced differing selective pressures associated with the evolution and loss of flight using DNA sequences from a total of 1476 nuclear single-copy orthologous protein-coding genes and 13 mitochondrial protein-coding genes obtained from transcriptomes. Firstly, we test for evidence of positive selection during the time when flight originated. Secondly, we test for positive and relaxed selection among multiple evolutionary losses of flight, which provide more recent and naturally replicated evidence for genes potentially associated with the evolution and maintenance of flight. In addition to using multiple evolutionary shifts in a biological or ecological trait to identify common genetic trends associated with that shift (e.g. [9][29]), we additionally use the reverse direction event to serve as comparison with the sole case of flight gain in hexapods. Thirdly, to examine further the relationship between energy-related genes and flight, we test for positive selection in available nuclear OXPHOS and mitochondrial OXPHOS genes throughout the major lineages of hexapods.

DATA DESCRIPTION

The nuclear genetic data used in this study consist of transcriptome-derived DNA sequences obtained as part of the 1000 Insect Transcriptome Evolution (1KITE) project (http://www.1kite.org) and additional hexapod genomes, as is presented in Misof et al. [2]. We utilized the current assembly version 2 (strict assembly followed by check for cross contamination, described in [30]) of transcript data of 101 species ([2], NCBI accession

PRJNA183205, individual accessions provided in supplementary data [31] Table S1) and assigned transcripts to 1476 single-copy nuclear orthologous genes included in the ortholog set published by Misof et al. [2]. We additionally included the 12 reference species with an official gene set available and used by Misof et al. [2] to infer orthology; thus, data for 113 species were available in total. Orthology assignment of transcripts, alignment, outlier check, alignment refinement, and generation of nucleotide alignments followed the guidelines described in Misof et al. [2] with some modifications (see Methods section). Sequences for the 13 mitochondrial protein-coding genes were obtained from the associated mitochondrial transcriptome sequencing project of BGI, with some substitution of sequences from mitochondrial genomes published on NCBI to increase completeness (species and sources of data provided in [31] Table S13). Mitochondrial sequences were aligned with EMBL-EBI Clustal Omega (Clustal Omega, RRID:SCR_001591) [32] and Pal2Nal [33]. Guidance [34] was applied to mask sequence regions that were unreliably aligned. The phylogenetic tree topology used here for selection tests was obtained from Misof et al. [2]. The data sets supporting the results of this article are available in the *Giga*DB repository [31].

ANALYSES

Positive selection associated with the origin of flight

Tests of positive selection were performed for each lineage of interest via branch-site models, which estimate dN/dS ratios at each codon site and between branches such that positive selection is detected in the lineage of interest if a subset of codon sites have dN/dS ratios greater than 1, while the other lineages have ratios less than 1 or equal to 1, indicating purifying selection or neutral evolution, respectively. Out of 954 nuclear genes tested in the lineage leading

to the pterygote insects (‘P’ in Figure 1), 126 (13%) were detected to be under positive selection; 39 of these were uniquely detected to be under positive selection in branch ‘P’ and not detected in either branch ‘U’ (upstream) or ‘D’ (downstream). The 39 unique candidate genes over-represented Gene Ontology categories related to ‘spliceosome’, ‘protein binding’, ‘protease’, and ‘RNA catabolic process’ (Table 1). The candidate gene list included *frayed (fray)* and *NADH dehydrogenase (ubiquinone) 23 kDa subunit (ND-23)*, related to wing development and the mitochondrial respiratory chain, respectively, but such functional categories did not contain an over-representation of genes exhibiting evidence of positive selection. When grouping multiple Gene Ontology terms of potential interest related to wing or mitochondrion/ATP-binding/OXPHOS-related functions, neither of these grouping were significantly over- or under-represented by the 39 candidate genes as compared to the non-candidate genes (wing: 2.6% in candidate list of genes displaying signature of positive selection vs. 3.0% in non-candidate list, $p_{\text{Fisher's Exact (1-tailed)}}=0.69$; mitochondrion-related: 15.8% vs. 17.4%, p=0.67; only over-representation p values shown). Out of 13 nuclear OXPHOS genes available in the background gene set of 954, only one was in the candidate list (2.6% vs. 1.3%, p=0.42). None of the 13 mitochondrial genes were detected to be under positive selection in the ‘P’ lineage after Benjamini-Hochberg correction. Gene names and descriptions for candidate and background genes for all analyses are provided in [31] Table S15.

Table 1. Positively selected genes in the lineage (‘P’) leading to Pterygota as over-represented in A) Gene Ontology (GO) categories from DAVID analysis and B) Biological Process categories from PANTHER analysis. Terms are for positively selected genes uniquely detected in the ‘P’ lineage and not in two control lineages tested (‘U’ and ‘D’). Categories with $p<0.05$ are shown;

full results are given in [31] Table S11. 954 background genes were mapped to A) 914 IDs and B) 894 IDs; 39 unique candidate genes were mapped to A) 38 IDs and B) 35 IDs. Statistical over-representation is tested by modified Fisher's exact tests in DAVID and binomial statistics in PANTHER, with raw p values provided here.

A) DAVID Gene Ontology results

	914 total genes	38 positively selected genes			
**GO term**	**# in category**	**Expected #**	**Observed #**	**P value**	**Fold enrichment**
precatalytic spliceosome	34	1.4	7	0.00084	5.0
mRNA splicing, via spliceosome	44	1.8	8	0.00087	4.4
catalytic step 2 spliceosome	30	1.2	6	0.0032	4.8
protein binding	81	3.4	8	0.035	2.4
Protease	19	0.8	4	0.038	5.1
mRNA processing	9	0.4	3	0.044	8.0

B) PANTHER Biological Process results

	894 total genes	35 positively selected genes			
**PANTHER GO-Slim Biological Process term**	**# in category**	**Expected #**	**Observed #**	**P value**	**Fold enrichment**
RNA catabolic process (GO:0006401)	9	0.4	2	0.048	5.7

Positive selection associated with flight loss

Eleven lineages (Figure 1) representing flight losses had between 0.8 and 53.7% of genes exhibiting positive selection, with a median of 2.4%. After considering the counts of genes detected under positive selection in the selected related flying lineages, 21 genes were still commonly (in three or more lineages) under positive selection in the flightless lineages. These genes over-represented Gene Ontology categories of 'coiled coil' (a protein structural motif), 'nucleus', and 'dendrite morphogenesis' (Table 2). When considering only the eight fully flightless lineages (excluding female flightless lineages) and seven selected related flying lineages, the Gene Ontology categories for the candidate genes were similar: including the three listed above, plus 'DNA binding', 'cytosol', and 'developmental protein', and process categories

additionally included ‘protein methylation’ ([31] Table S11). These 17 genes did not over- or under-represent gene category descriptions relating to wings or mitochondrion/ATP-binding/OXPHOS-related functions, with the wing-related genes being *absent, small, or homeotic discs 2 (ash2)* and *no ocelli (noc)* (11.8% in candidate list vs. 3.1% in non-candidate, p=0.099) and two ATP-linked genes including *gluon(glu)* (11.8% vs. 17.2%, p=0.82). No nuclear OXPHOS genes were present in the 17-gene candidate list among the 13 nuclear OXPHOS genes tested (p=1.0).

Table 2. Genes detected to be under positive selection in three or more lineages with flight loss as over-represented in A) Gene Ontology (GO) categories from DAVID analysis and B) Biological Process categories from PANTHER analysis. Counts of positively selected genes in related flying lineages were removed from counts in flightless lineages to determine candidate genes before functional analysis. In B) child (sub-categorical) processes are indented below parent processes. Categories with p<0.05 are shown; full results are given in [31] Table S11. 1284 total background genes were mapped to A) 1229 IDs and B) 1207 IDs; 21 candidate genes were mapped to 21 IDs (A and B).

A) DAVID Gene Ontology results

	1229 total genes	21 candidate positively selected genes			
**GO Term**	**# in category**	**Expected #**	**Observed #**	**P value**	**Fold enrichment**
coiled coil	223	3.8	9	0.018	2.4
nucleus	269	4.6	9	0.048	2.0
dendrite morphogenesis	21	0.4	3	0.050	8.4

B) PANTHER Biological Process results

	1207 total genes	21 positively selected genes			
**PANTHER GO-Slim Biological Process term**	**# in category**	**Expected #**	**Observed #**	**P value**	**Fold enrichment**
cellular component organization	113	2.0	5	0.041	2.5
organelle organization	64	1.1	4	0.0229	3.6
chromatin organization	18	0.3	2	0.0387	6.4

Relaxed selection associated with flight loss

Postulated relaxed selection was detected by increased dN/dS ratios across the fully-flightless vs. flight-capable branches of the tree (i.e. pooling branches by flight state) calculated for the entire length of each gene tested, as opposed to positive selection which was detected using branch-site models (accounting for dN/dS ratios at each site) on individual lineages of interest. Fifty-six out of 1285 nuclear genes tested show significantly higher ($p<0.05$) dN/dS ratios in the flightless pterygote lineages than in related flying lineages (red vs. blue lineages in Figure 1). None of the 56 candidate genes overlapped with the 17 genes detected as candidates in the positive selection analysis of fully flightless lineages. The main GO categories were related to 'spliceosome', while processes were 'RNA localization', 'negative regulation of apoptotic processes', and 'extracellular transport' (Table 3). The candidate gene descriptions contained wing-related functions in genes that included *tankyrase (Tnks)*, and wing- and ATP-binding related functions in the gene *tricornered (trc)*. Neither the wing-related nor mitochondrion/ATP-binding/OXPHOS-related groupings were over- or under-represented compared to the non-candidate genes (wing: 3.7 vs. 3.4%, p=0.56; mitochondrion: 13.0% vs. 17.9%, p=0.87). None of the nuclear OXPHOS genes were present in the candidate gene set out of 14 nuclear OXPHOS genes tested (p=1.0). Only two nuclear OXPHOS genes had higher dN/dS ratios in flightless lineages, with 12 showing higher dN/dS ratios in flying lineages (12 out of 14, $p_{binomial}=0.013$), and four of those exhibited a significant difference ([31] Table S9). The *myosin binding subunit (Mbs)* gene ($p=1.0x10^{-16}$) and IDH gene (p=0.050) showed higher dN/dS ratios in flying than flightless lineages. The mitochondrial genes showed significantly higher dN/dS ratios in the flightless pterygote lineages, which here included both-sexes-flightless and female-flightless

lineages, than in the related flying lineages (Figure 2). Eleven out of 13 mitochondrial OXPHOS genes ($p_{binomial}$ = 0.023), and all five of the genes exhibiting a significant difference, had higher dN/dS ratios in the flightless lineage than in the related flying lineage (p values given in [31] Table S10).

Table 3. Genes detected to be under relaxed selection (higher dN/dS ratios) in flightless pterygote lineages as compared to related flying lineages as over-represented in A) Gene Ontology categories from DAVID analysis and B) Biological Process categories from PANTHER analysis. In B) child (sub-categorical) processes are indented below parent processes. Categories with p<0.05 are shown; full results are given in [31] Table S11. 1285 total background genes were mapped to A) 1231 IDs and B) 1209 IDs; 56 candidate genes were mapped to A) 54 IDs and B) 53 IDs.

A) DAVID Gene Ontology results

	1231 total genes	54 higher dN/dS genes			
**GO Term**	**# in category**	**Expected #**	**Observed #**	**P value**	**Fold enrichment**
mRNA splicing, via spliceosome	50	2.2	8	0.0069	3.6
catalytic step 2 spliceosome	35	1.5	6	0.021	3.9
precatalytic spliceosome	39	1.7	6	0.033	3.5

B) PANTHER Biological Process results

	1209 total genes	53 higher dN/dS genes			
**PANTHER GO-Slim Biological Process term**	**# in category**	**Expected #**	**Observed #**	**P value**	**Fold enrichment**
RNA localization	11	0.5	3	0.013	6.2
death	13	0.6	3	0.020	5.3
cell death	13	0.6	3	0.020	5.3
apoptotic process	13	0.6	3	0.020	5.3
negative regulation of apoptotic process	1	0.04	1	0.043	22.8
localization	144	6.3	12	0.020	1.9
extracellular transport	1	0.04	1	0.043	22.8

Overlap between positive and relaxed selection results

Three biological process categories overlapped between the positive selection analyses from the Pterygota lineage (39 candidate genes) and the relaxed selection analyses in flightless vs. flying lineages (56 candidate genes): ‘mRNA splicing, via spliceosome’, ‘catalytic step 2 spliceosome’, and ‘precatalytic spliceosome’. Two genes overlapped between these candidate lists of genes under positive or relaxed selection, out of 933 genes in common between the two sets of tests: *hephaestus(heph)* and *Ribosomal protein L13A(RpL13A)*, together belonging to the DAVID functional annotation term ‘mRNA binding’ (p_{DAVID}=0.035) ([31] Table S11).

Positive selection in nuclear and mitochondrial OXPHOS genes in hexapod lineages

Six of the 14 nuclear OXPHOS genes present in the total gene set exhibited positive selection in at least one branch (tree with one species chosen per order, represented in Figure 3), along with four of 10 nuclear genes that were randomly selected to use as point of comparison, and three of the five other nuclear genes chosen *a priori* (genes listed in [31] Table S12). Each mitochondrial OXPHOS gene had positive selection detected in at least one branch in either the 32-species tree with one species selected per order (Figure 3) or the 66-species tree with multiple species selected per order (results in [31] Table S13). The apterygote lineages (i.e. primarily flightless lineages, highlighted in grey) as well as lineages in orders Odonata (i.e. dragonflies and damselflies) and Ephemeroptera (i.e. mayflies), which have a direct flight mechanism, did not exhibit many signatures of positive selection, excepting in Protura and the interior branch leading to Protura + Collembola (Figure 3). In the mitochondrial tree including more than one species per order, again no positive selection was detected in apterygotes (excepting in Protura), but

some instances of positive selection were revealed within the Odonata + Ephemeroptera clade ([31] Table S13). Positive selection in mitochondrial OXPHOS genes was more common in the holometabolous (i.e. complete metamorphosis) insect clade (labeled 'H' in Figure 3) than in the polyneopteran clade (labeled 'L' in Figure 3); both of those clades contain a similar number of orders and are of similar age (approximately 362 and 387 million years old, respectively [2]). Nuclear genes showed little difference in the prevalence of positive selection between holometabolous and polyneopteran clades (eight vs. seven instances).

DISCUSSION

This study tested for trends in the categories of genes evolving under differing selective pressures associated with flight evolution and loss in hexapods. The incorporation of both transition directions allows a comparison of trends in the genes under adaptive evolution and relaxed selective constraints with the evolution and loss of flight, respectively. We observed the origin of Pterygota to be associated with significant signatures of positive selection in categories of genes tied to catabolic processes and spliceosome, the latter overlapping with gene categories represented by relaxed selection tests in lineages having undergone flight loss. Flight loss was also accompanied by positive selection in various categories of genes. These tests did not reveal any significant selection pressures in nuclear energy-related genes associated with flight evolution and loss, while mitochondrial genes displayed trends in line with previous expectations of relaxed selection associated with flight loss [9][17][18]. The holometabolous insects had the highest prevalence of positive selection.

OXPHOS genes related to flight: a priori gene selection

Energy-related genes, specifically mitochondrial and to a lesser extent nuclear OXPHOS genes, were expected to show signatures of positive selection with the origin of active flight and relaxed selection with the loss of flight. In a study of bat flight evolution [10], the lineage leading to bats was associated with 23% of mitochondrial-encoded OXPHOS genes displaying positive selection, while positive selection was only 3% more common in bat nuclear OXPHOS genes than in the lineage leading to rodents; other mitochondrial-associated nuclear genes showed no difference between lineages. In our study, no positive selection was observed associated with the origin of Pterygota for the mitochondrial OXPHOS genes, and no over-representation of positive selection was observed for nuclear OXPHOS genes as compared with the background gene sets or with other deep branches ("U" and "D"). It is possible that some signatures of selection were too difficult to detect due to the long time frames, given the trends in mitochondrial and nuclear OXPHOS genes in other insect [17], bird [9], and bat [10] taxa that have evolved or lost flight more recently. The origin of flight in Pterygota occurred approximately 400 million years ago, while bats originated about 60 million years ago [35].

Associated with flight loss, nuclear OXPHOS genes surprisingly showed more often higher dN/dS ratios in flying than flightless lineages (with 4 genes having significant differences), which was contrary to expectations when testing for relaxed selection. However, mitochondrial OXPHOS genes showed evidence of relaxed selection in flightless as compared with flying lineages as demonstrated by significantly higher dN/dS ratios in flightless lineages. This is in accordance with previous observations of proposed relaxed selection in mitochondrial genes associated with flight loss within insect orders [17] and in birds [9]. These findings also mirror patterns of molecular evolution in weakly vs. highly locomotive fish [36] and mammals

[9]. Four out of the five significant differences in dN/dS ratios between flightless vs. flying insect lineages were observed in the mitochondrial cytochrome genes (COI, COII, COIII, CytB), while only one significant difference was present for the other mitochondrial OXPHOS genes. These differences among genes could stem from varying levels of purifying selection. dN/dS ratios of mitochondrial protein-coding genes in mammals suggest the greatest purifying selection on sequences of COI, COII, COIII, and CytB [37], while in beetles the lowest rates of substitutions at 1st and 2nd codon positions were observed in COI, CytB, ND1, COIII, and COII [38]. Thus, the trends between flightless vs. flying lineages in their COI, COII, COIII, and CytB genes could be due to greater purifying selection on those genes, in general, thus allowing the effect of relaxed selection with flight loss to become apparent.

Previously, mitochondrial OXPHOS genes were examined for positive selection throughout a variety of phylogenetic lineages in insects, and there were fewer signatures of selection detected in apterygote lineages [18]. Here, we included all extant currently recognized insect orders, improving on the representation of both apterygote hexapod lineages (five orders as compared to two included in Yang et al. [18]) and pterygote lineages (27 orders as compared to 20). As well, we examined nuclear OXPHOS genes. We similarly observed a lack of positive selection in apterygote lineages, and nuclear OXPHOS genes were not disproportionately evolving under positive selection specifically associated with the origin of Pterygota. Mitochondrial OXPHOS genes exhibited substantial positive selection in the holometabolous insects, while nuclear OXPHOS genes showed little proportional difference in comparison to the control genes. Although the number of taxa included here for holometabolous insects (clade 'H" in Figure 3) was similar to that for the polyneopteran clade ('L'), the holometabolous insects represent 83% of all insect species [39]. The detection of selection may in part be linked to the

speciation rate of the group, since species diversity and molecular evolutionary rates have been observed to correspond (e.g. [40]). However, this potential mechanism does not fully explain the findings as several highly species-rich groups (such as Lepidoptera) did not exhibit significant positive selection.

It was previously proposed that the type of flight mechanism—asynchronous vs. synchronous flight—may explain trends in adaptive molecular evolution in flying insects [18]. Asynchronous flight, the ability for multiple wing beats per nerve impulse, is present for all of Hymenoptera (i.e. bees, wasps, ants, sawflies), Coleoptera (i.e. beetles), Strepsiptera (i.e. twisted-wing parasites), Diptera (i.e. flies), and Thysanoptera (i.e. thrips) [41]. However, these mechanisms may have similar energetic costs; although synchronous flight may cost more metabolically per stroke, asynchronous fliers often achieve higher stroke frequencies [42][43]. The pattern of positive selection here does not mirror the occurrence of asynchronous vs. synchronous flight. Positive selection associated with the origin of Pterygota was not greater than in downstream lineages. The origin of flight may have set the stage for downstream selection pressures within some lineages related to metabolic efficiency. However, other factors could also be influencing detection of positive selection in particular orders, such as fast mitochondrial gene substitution rates in Strepsiptera [44], proposed to be due to the transition to parasitism. The trend in holometabolous insects may, in general, relate to other biological traits tied to holometaboly itself, such as the occurrence of rapid development, which is thought to constrain genome size in that group [45]. Overall, the pterygotes have a greater prevalence of positive selection in OXPHOS (especially mitochondrial) genes than the apterygotes, as was expected tied to flight ability, with no apparent correspondence to any single flight-related mechanism.

Exploratory analysis of gene categories

In this exploratory analysis, we observed the origin of Pterygota to be associated with signatures of positive selection in protease and RNA catabolic processes genes, whose categories have a common theme of catabolism, which is the subset of metabolic activities involved in breaking down molecules to release energy and building components. Spliceosome-related genes were also overrepresented in the positive selection results. The origin of Pterygota is associated with additional apomorphies other than flight, such as the evolution of metamorphosis and direct sperm transfer; as such, it is possible that results relate to functions other than flight or wings. The fit of Gene Ontology categories with biological expectations would not validate the selection results as it is possible to create a biological narrative from inaccurate results through over-interpretation [46]. However, interestingly, categories ‘proteasome’ and ‘spliceosome’ were also observed to be more highly expressed in flying vs. flightless morphs of aphids [21].

Associated with flight loss, the gene categories exhibiting signatures of relaxed selection also frequently included ‘splicing’ or ‘spliceosome’. The mirrored occurrence of this category between flight gain and loss suggests a biological association with flight in insects. While one transcription study has linked expression levels of spliceosome-related genes to flying vs. flightless morphs of cotton aphids [21], citrus and pea aphids do not exhibit a major difference in this category between flying and flightless morphs [22][47], and expression differences in this category are only associated with sex-related differences within flying morphs in the brown planthopper [48]. ‘Localization’ was also found to be a general category under relaxed selection in flightless insects, which mirrors the observation of over-representation of expression in the localization category between winged vs. unwinged morphs of pea aphids [22].

Alternative splicing of exons in pre-mRNAs is one mechanism that contributes to increased phenotypic complexity [49], and as such, directional selection on splicing mechanisms may be congruent with the evolution of a complex trait such as flight ability. Alternative splicing is directly necessary for insect flight, which could account for splicing-related genes being under relaxed selection with flight loss as well. Almost all structural molecules in insect flight muscles, such as proteins and RNAs, exist as multiple isoforms [50]. Alternative splicing allows various isoforms of muscle-related molecules and as such appears to be an important mechanism to allow quantitative adjustment of muscle force and power output [50][51]. However, it is unclear whether alternative splicing is more frequently occurring for these flight-related genes than all genes in general, as alterative splicing has been observed to occur in a large proportion of genes, at least in humans, including estimates of around 95% of multi-exon genes [52]. In addition, in multiple studies of flying vs. flightless morphs of insects, there are no significant differences in expression levels of splicing genes, suggesting no large difference in general occurrence of splicing in flying vs. flightless insects. However, flightless vs. flying morphs of insects do not represent evolutionarily distinct lineages, and so genes exhibiting different expression levels among morphs may not be those bearing signatures of differences in selection regime between flightless vs. flying insects on much longer evolutionary timescales. Thus, we suggest genes related to splicing are a potential category for further investigation of whether differing selection pressures occurred with the origination of flight and flight loss in insects. This study examined only coding regions and was not able to consider changes in gene regulatory regions, which affect co-regulation. Co-regulation is important in processes including energy production [53]. Our results that suggest alternative splicing is an important gene functional category for flight

evolution may be a symptom of the involvement of regulatory changes in general, which we were not able to test here.

The loss of flight is not only associated with the change in flight ability, but also major changes in ecology and life history, such as diet, predation, habitat (e.g. woodlands, deserts), courtship, and often reduction in dispersal ability [8][17]. Such changes are specific to certain species or clades, and thus the use of multiple lineages may help to eliminate some noise created by confounding biological or ecological factors. Nonetheless, some associated factors, such as reduced dispersal ability, are likely commonly associated with flight loss, and therefore, the results here are likely impacted by co-occurring factors in addition to change in flight capability itself. Categories of genes under positive selection associated with flight loss included protein motif (coiled coil), the nucleus, dendrite morphogenesis, and chromatin organization. These do not clearly fit with more highly expressed gene categories in flightless vs. flying morphs of insect species observed by expression studies. For example, genes potentially undergoing positive selection with flight loss could be tied to sugar metabolism [47] or reproduction, such as vitellogenin (an egg yolk protein precursor) [48]. Due to the generally reduced dispersal ability associated with flight loss and the energy trade-off between dispersal and reproduction [22], we expected positive selection in genes or processes tied to fecundity. For a gene to show signals of positive selection in multiple flight loss examples, the same ortholog must have adaptively diverged from flight-capable lineages. Given the long evolutionary history in the flight-adapted state before flight losses occurred and the seeming ease in which flight ability can be 'turned off' developmentally by loss of function of specific genes [54], the relaxed selection tests may be more able to uncover trends in genes associated with flight loss than positive selection. Even so, given the consistent association between flight loss and increased reproductive ability, future

studies using more genomic information may uncover positive selection with flight loss that we did not, or were not able to, detect here.

Caveats and next steps

The detection of positive selection can be affected by many factors including quality of the sequence alignment [55] and false positives and negatives associated with level of substitution saturation [56]. This study involved investigating positive and relaxed selection along longer timespans than is typical in genome-wide scan studies (e.g. approximately 60 million years separating dolphin vs. cow [57]). Thus, it is likely that positive or relaxed selection could be difficult to detect due to long time frames and various periods of positive and purifying selection, especially in the lineage leading to Pterygota. While Gene Ontology categories are useful to look for trends in genomic selection, different gene categories could be detected under positive selection with varying species choice, change in background genes available, the Gene Ontology tool [58], or version of tool applied.

The replication provided by multiple losses of flight can help to narrow down uncertainty due to taxon selection and analysis methods, also helping to illuminate the interpretation of the molecular signatures associated with the single evolution of flight. Despite the long timeframes included here, the trends observed for dN/dS ratios in flightless lineages as compared to flying lineages are similar to trends observed on shorter timeframes within insect orders [17] and other animal taxa [9]. Future insect phylogenomic work with increased taxonomic sampling would allow further improvement in the number of cases of flight loss available, with increased accuracy of the phylogenetic mapping of transitions in flight state. Additionally, with better taxonomic sampling, the effects of co-occurring confounding factors (e.g. parasitism) could be

separated, and trends for each type of flight loss (e.g. female flightlessness vs. full flight loss) could be further investigated.

Importantly, expansion of the loci included in analysis would provide further insight into selection associated with flight gain and loss in insects. The single-copy, transcriptome-derived genes analyzed here represent a portion of all protein-coding genes in the insect genomes and thus restricted the total pool of possible gene categories that could be detected under differing selection pressures; for example, around 16,000 total genes are observed in *Drosophila* species [59]. Many gene functional categories are poorly represented in our data set, and thus the "expected" counts are low in some categories. The results of this study might therefore be considered hypotheses for testing using a larger portion of genomes in future studies. The orthologous genes included here represent those more essential for life as they are present and transcribed across a range of arthropod species, life stages, and sexes; many serve basic cellular functions [2]. Thus, genes with more specialized functions, including some related to the development of wings or flying, are not represented. Furthermore, there may be important changes in regulatory (non-protein coding) regions, which govern expression levels and the specific tissues in which expression occurs, associated with flight and flight loss. Thus, future comparative genomics analysis using DNA-derived genomes could investigate both protein-coding and non-coding loci, as well as use full genomic data to assess gene gains or losses. Investigation of gene families would likely prove interesting, given that other studies have provided evidence for trends in adaptation based on gene presence and absence or gene family evolution, such as diversification among paralogous genes [59][60].

Conclusions

This study presents an exploratory examination of the genes under positive and relaxed selection associated with the evolution and loss of flight in insects. Considering this study together with prior studies on other animal groups [9][10][17][18], similarities were detected in the selection regime acting upon mitochondrial genes across multiple flying vs. flightless animal groups. These results indicate convergent trends in molecular evolution that parallel convergent functional evolution in evolutionarily disparate animals. Various nuclear gene categories were linked to flight evolution and loss, which could be further explored for potential biological significance. Intriguingly, we found mirror-image patterns of selection in genes relating to splicing: positive selection with the origin of Pterygota and relaxed selection in flightless lineages. The results here contribute insight into the evolution of an important and unique trait that has played a major role in shaping the diversity of life.

METHODS

Genetic data

Generation of the nuclear gene nucleotide alignments from the transcripts included these steps: 1) orthologous transcripts for each species were assigned to 1476 single-copy orthologous genes using an early version of Orthograph [61], version 0.5.4 (available from Github: https://mptrsen.github.io/Orthograph/); 2) each gene was aligned with MAFFT v7.017 (MAFFT, RRID:SCR_011811) [62] using the L-INS-I algorithm for amino acid sequences translated from original nucleotide transcripts during orthology assignment; 3) multiple sequence alignment of each orthologous gene was refined by identification of outlier sequences; refinement of outliers

was performed using a profile alignment approach with MAFFT L-INS-I --add; the alignment was again checked for remaining outliers; final removal of outliers was performed; and 4) a modified version (see [2]) of Pal2Nal [33] was applied to obtain the corresponding nucleotide multiple sequence alignments using the protein alignments as blueprint.

Exploratory test of positive selection in lineage leading to Pterygota

Twenty-eight hexapod species were selected to maximize the number of shared nuclear genes available for analysis as well as the phylogenetic representation of pterygotes and non-pterygote hexapods. Not all genes were available for all species in the candidate alignments, and thus species were selected with the trade-off of number of species versus obtaining the largest gene set. For this test, we excluded flightless species or orders from within Pterygota, i.e. representing secondary flight losses. Species selection was performed in a phylogenetically stratified way, with the final list of 28 species being those that gave the maximum gene count: 1) all five apterygote orders were included, with a maximum of three species per order, but allowing up to one missing sequence per gene for this set; 2) one species from Odonata and one species from Ephemeroptera were included, with no missing sequences allowed; 3) one species per each of five orders of Polyneoptera was included, allowing one missing sequence per gene for this set; 4) one species from each of 10 orders in the clade including Thysanoptera and Diptera (Fig. 1) was included, allowing up to three missing sequences per gene (species selected shown in Fig. 1). This resulted in 954 genes out of 1476. Similarly, 27 species representing apterygote and pterygote hexapod orders were selected for the 13 mitochondrial protein-coding genes, with no missing sequences allowed.

We tested for evidence of positive selection in these nuclear and mitochondrial genes in the lineage leading to Pterygota (Fig. 1 branch ‘P’). We used the branch-site method of detecting positive selection [63] in the program PAML *codeml* version 4.8 (PAML, RRID:SCR_014932) [64], with the fit of models A1 (non-synonymous-to-synonymous (dN/dS) ratio fixed at 1) vs. A (dN/dS ratio free to vary) (each model with four classes of sites, each class allowing a certain combination of dN/dS ratios representing positive selection, purifying selection, or neutral evolution) compared for each gene separately through likelihood ratio tests [65]. For this and subsequent analyses, we corrected for false discovery due to multiple genes being tested by using the Benjamini-Hochberg correction [66] for each gene within a set, with a family-wise alpha of 0.05.

We repeated the tests on two additional lineages to serve as a null hypothesis to compare to the results for the lineage ‘P’. Branch ‘U’ (upstream) and ‘D’ (downstream) (Fig. 1) were tested. Using these results, we separated out genes that were uniquely detected as being under positive selection in the lineage leading to pterygote insects. These unique genes were subjected to Gene Ontology (GO) analysis, described in the ‘Functional analysis’ section below.

Exploring genes under positive selection with flight loss

Eleven cases of flight loss were identified by mapping flight state on the available phylogenetic tree (Fig. 1) adopted from Misof et al. [2], and three of these cases involved flight loss only in the female sex. Not all of these evolutionary losses were accurately mapped to the correct branch here, given the available species sampled. For example, a loss may have occurred in the common ancestor of a family, but only species representing superfamily-level divergences were available for our analysis. In the case of phasmids, flight loss occurred multiple times

within the order [7][67]. However, all available species were flightless, and thus the losses could not be represented accurately on the phylogeny; we tested the branch leading to the phasmid clade to approximate the timing of early flight losses in that order. Due to incomplete phylogenetic mapping of some of the flight loss events, the branches tested here likely represent some flying lineage history in addition to flightless lineage history, which may cause underestimation of molecular signal due to flight loss. A qualitative assessment is provided to indicate the likely degree of accuracy in the mapping of each case of flight loss, considering the density of taxonomic sampling in that group and how frequently flight is thought to have been lost in those groups (Fig. 1 and [31] Table S4). Sub-trees including the lineage of interest, sister lineage(s), and three successively branching outgroups were used to test for signatures of positive selection associated with each case of flight loss separately in order to maximize gene coverage; no missing gene data were allowed for the species within each sub-tree. Each sub-tree contained 14 to 19 species, with 584 to 1174 genes available for all species in each analysis (listed in [31] Table S4). A total of 1284 genes was included, considering all 11 sub-trees.

A test for positive selection was performed on each of the 11 branches of interest for each sub-tree and gene separately. Those genes with significant p values (at 0.05 level after Benjamini-Hochberg correction) within a sub-tree were included in further analysis. We identified genes that were detected as evolving under positive selection in three or more of the 11 lineages tested. However, in order to eliminate those genes exhibiting a signature of selection in many lineages regardless of flight state, we also tested nine flight-capable lineages that were sister lineages or were closely related to the flightless lineages for positive selection using the same sub-trees as the flightless lineages (trees and results in [31]). There were numerous flight loss events in one sub-tree, and so there were fewer related flight-capable lineages to include,

resulting in nine flight-capable lineages tested overall (as compared with 11 flightless lineages). The counts of genes exhibiting a significant signature of positive selection ($p<0.05$ after Benjamini-Hochberg correction) were tallied for the flying lineages, and these counts were subtracted from the list of candidate genes for the flightless lineages. Those remaining genes with three or more counts of positive selection in the flightless lineages were included in functional analysis. This procedure was repeated for the eight cases of full flight loss (i.e. excluding the three cases of female-only flight loss) as compared to seven related flying lineages.

Exploring genes under relaxed selection with flight loss

Nuclear and mitochondrial genes were examined for relaxed selection associated with flight loss using branch models in PAML *codeml* to estimate dN/dS ratios for lineages of interest. For nuclear genes, the total 113-species tree (Fig. 1) was used, and missing data were allowed. Only genes with data for 80 or more species were included, resulting in 1285 genes tested. Flightless lineages representing full flight loss (not female-only flight loss) were coded one branch rate (red branches in Fig. 1) and the sister or related flight-capable lineages of similar tip number and taxonomic rank were coded together a separate rate (blue branches in Fig. 1), while all other lineages were coded as the background rate.

For each gene, a change in selection regime associated with loss of flight was concluded when there was a significantly increased dN/dS ratio (between 0 and 1) in flightless lineages as compared to flying lineages. Likelihood ratio tests between 3-rate trees (flightless [red], flying [blue], background [black + purple]) and 2-rate trees (flightless [red] + flying [blue] branches vs. all other lineages [black + purple]) were used to test for significant dN/dS differences between target lineages and sister lineages. P values were corrected by Benjamini-Hochberg correction

across genes, with a family-wise α = 0.05. Those genes that had a significantly higher dN/dS ratio in the flightless than flying lineages were examined by functional analysis (below) as compared to the total gene set tested. We interpreted increased dN/dS ratios as signifying relaxed selection. This interpretation of the dN/dS ratios involves the assumption that the majority of non-synonymous changes across a whole gene sequence are selectively neutral or slightly deleterious; by contrast, positive selection is assumed to affect a small minority of sites at which mutations with beneficial effect have occurred [68]. However, given that increased dN/dS ratios can be due to strong positive selection rather than relaxed selection (or in combination, in different parts of the gene), as a precaution we verified whether any genes from this list overlapped with those in the final candidate list for genes under positive selection in both-sexes-flightless lineages.

For mitochondrial genes, a 66-species tree adopted from Misof et al. [2] (similar to Fig. 1) was used, given in [31] S10 tree file. Since there is no ‘background’ gene set due to all mitochondrial genes being energy related, we directly compared the dN/dS ratios in the flightless vs. related flying lineages. In preliminary tests on these mitochondrial genes and in Mitterboeck and Adamowicz [17], the female-flightless lineages yielded similar results to full-flightless lineages as compared with related flying lineages. Due to this, and the smaller number of flightless lineages in the mitochondrial gene tree, we considered both female- and both-sexes flightless lineages in the flightless category (e.g. red + purple flightless lineages vs. blue flying lineages, with the black lineages all coded to the background rate).

Functional analysis

We tested for over-representation in Gene Ontology (GO) categories by the genes exhibiting positive or relaxed selection as compared to each total gene set analyzed ('background genes') using the DAVID version 6.8, October 2016 (Database for Annotation, Visualization and Integrated Discovery, RRID:SCR_003033) Functional Annotation chart tool [69][70] to identify enriched annotation terms and similarly using PANTHER (Protein Analysis Through Evolutionary Relationships) version 11.1 (PANTHER, RRID:SCR_004869) [71] to identify 'processes'. The genes were matched to *Drosophila* genome functional annotations where available, using the FlyBase ID (http://flybase.org/) for each gene. No additional false discovery rate correction was applied (p values are raw) as correction was already applied for the positive selection analysis, and also the number of candidate genes was lower than the optimal working input for DAVID (hundreds to thousands of genes [69]). For DAVID analysis, the expected number of genes in each GO category was calculated by the number of genes in the category divided by the number of background genes, multiplied by the number of candidate genes (e.g. expected number in Table 1 first GO term; (34/914)*38 = 1.4). The fold enrichment was calculated by the observed number divided by the expected number of genes in that GO term. The 1476 source genes used in our analysis are not representative of gene categories in the full insect genomes; we provide information on the gene categories over- or under-represented by these 1476 genes in relation to the full genome of *Drosophila melanogaster* in [31] Table S14. Our tests for over-representation of genes under positive or relaxed selection are in relation to each of our available gene sets, i.e. 'background' sets that are each a subset of the total 1476 genes. In addition to GO analysis, which provides information on functional terms based on over-representation available in the candidate gene set, we grouped lists of genes in similar DAVID terms (those terms present with default settings in the 'chart' or 'cluster' mode) existing

in our background 1476 gene set. The grouped terms were those related to each 1) wing development (4 chart and 1 cluster term for a total of 48 genes) and 2) mitochondrion/ATP-binding/respiratory-chain-related functions (7 chart and 11 cluster terms for a total of 237 genes). We acknowledge that these groupings do not include all possible genes related to the wing or mitochondrion-related functions of interest in the dataset but provide two larger, functionally-defined groupings to test. Grouping similar GO terms for analysis can improve interpretation of results by increasing statistical power that is diminished by the dependence between GO terms, thus revealing trends not detected in individual GO terms [72]. We test for over- and under-representation of these gene sets in candidate vs. non-candidate lists using 1-tailed Fisher's exact tests in R version 3.3.1 [73] ([31] Table S16). As well, we report whether nuclear OXPHOS genes were over- or under-represented in the candidate gene sets, with genes identified through names provided in Tripoli et al. [14].

Positive selection in energy-related genes in Hexapoda

Specific genes were investigated that related to energy production or were *a priori* hypothesized to be related to flying or flight loss. These included 14 nuclear OXPHOS genes available in the total gene set (1476 genes) identified via their FlyBase IDs, which are a subset of the 78 nuclear OXPHOS genes listed in Tripoli et al. [14], and five other genes of interest identified by name or description in DAVID functional annotation: *wingless*, *IDH*, *flightless1*, *myosin binding subunit*, and an energy-related gene (Dmel_CG1271). Ten additional genes with full species coverage were pseudo-randomly selected (not considering function, with the selections spread out by FlyBase IDs) and also analyzed to check for phylogenetic biases in the positive selection results. We selected one species per hexapod order (32 orders) and one species

from each of two arthropod outgroups (outgroups were available for the nuclear genes only; 34 species total), for each set of nuclear and mitochondrial genes. In selecting species, we considered gene completeness, with preference for those species available across the most genes of interest. In a few cases, substitutions of some species were made to improve gene completeness (species lists provided in [31] Table S12). Mitochondrial genes, where gene sampling was more complete for species, were additionally tested with more than one species per order (up to 6 species) to investigate effects of species sampling on the results; the 66-species tree was the same as that used for relaxed selection analysis of mitochondrial genes (in [31] Table S10). Tests for positive selection were conducted on all lineages using the program HyPhy [74] and the Branch-site REL (Random Effects Likelihood) model [75] implemented on the publically-available DataMonkey server (Data Monkey, RRID:SCR_010278) [76].

Abbreviations

GO: Gene Ontology; PAML: Phylogenetic Analysis by Maximum Likelihood

Acknowledgements

We thank Lili Zhou for her contribution during the early stages of this project. We give a huge thank you to the 1KITE community, who have made the data possible, especially Alexander Donath and Lars Podsiadlowski, who worked on the current transcriptome assembly, cross contamination check, and submission to NCBI. We thank Stephen Marshall and Daniel Ashlock for input on the ideas in earlier versions of this manuscript and Jim Marden and Christopher Jones for valuable input on the manuscript as reviewers.

Funding

This work was supported by the University of Guelph (Integrative Biology PhD Award, Dean's Tri-council Scholarship, and Ontario Graduate Fellowship to T.F.M.), the Government of Ontario (Ontario Graduate Fellowship to T.F.M.), and by the Natural Sciences and Engineering Research Council of Canada (Alexander Graham Bell Canada Graduate Scholarship to T.F.M., Discovery Grants 386591-2010 to S.J.A. and 400479 to J.F.). X.Z. is supported by the China Agricultural University through the Chinese Universities Scientific Fund (2017QC114).

Availability of data and materials

The datasets supporting the results of this article are available in the *GigaDB* repository associated with this publication [31], including input and output information such as gene lists, newick trees, p values for selection tests, and functional analysis results. The nuclear sequence data are available associated with the NCBI Project PRJNA183205.

Authors' contributions

Conceived or designed work: X.Z., T.F.M., R.Z., W.S., J.F., S.J.A., S.L. Filtered genetic data: K.M., S.L. Designed data sets and analyses: T.F.M., S.L. Conducted bioinformatics for PAML analyses: S.L. Conducted Gene Ontology and HyPhy analyses: T.F.M. Drafted the article, generated figures and tables: T.F.M. Revised article drafts: T.F.M., S.J.A., S.L., J.F., K.M. All authors have read and approved the final manuscript.

Competing interests

The authors declare that they have no competing interests.

REFERENCES

1. Mayhew PJ. Why are there so many insect species? Perspectives from fossils and phylogenies. Biol Rev. 2007;82:425–54.

2. Misof B, Liu S, Meusemann K, Peters RS, Donath A, Mayer C, et al. Phylogenomics resolves the timing and pattern of insect evolution. Science. 2014;346:763-767.

3. Averof M, Cohen SM. Evolutionary origin of insect wings from ancestral gills. Nature. 1997;385:627–30.

4. Clark-Hachtel CM, Linz DM, Tomoyasu Y. Insights into insect wing origin provided by functional analysis of vestigial in the red flour beetle, *Tribolium castaneum*. Proc Natl Acad Sci USA. 2013;110:16951–6.

5. Medved V, Marden JH, Fescemyer HW, Der JP, Liu J, Mahfooz N, et al. Origin and diversification of wings: insights from a neopteran insect. Proc Natl Acad Sci USA. 2015;112:15946–51.

6. Grimaldi D, Engel MS. Evolution of the Insects. New York: Cambridge University Press; 2005.

7. Whiting MF, Bradler S, Maxwell T. Loss and recovery of wings in stick insects. Nature. 2003;421:264–7.

8. Roff DA. The evolution of flightlessness in insects. Ecol Monogr. 1990;60:389–421.

9. Shen Y-Y, Shi P, Sun Y-B, Zhang Y-P. Relaxation of selective constraints on avian mitochondrial DNA following the degeneration of flight ability. Genome Res. 2009;19:1760–5.

10. Shen Y-Y, Liang L, Zhu Z-H, Zhou W-P, Irwin DM, Zhang Y-P. Adaptive evolution of energy metabolism genes and the origin of flight in bats. Proc Natl Acad Sci USA. 2010;107:8666–71.

11. Roff DA. Life history consequences of bioenergetic and biomechanical constraints on migration. Am Zool. 1991;31:205–15.

12. Krogh A, Weis-Fogh T. The respiratory exchange of the desert locust (*Schistocerca gregaria*) before, during and after flight. J Exp Biol. 1951;28:344–57.

13. Erecinska M, Wilson DF. Regulation of cellular energy metabolism. J Membr Biol. 1982;70:1–14.

14. Tripoli G, D'Elia D, Barsanti P, Caggese C. Comparison of the oxidative phosphorylation (OXPHOS) nuclear genes in the genomes of *Drosophila melanogaster*, *Drosophila pseudoobscura* and *Anopheles gambiae*. Genome Biol. 2005;6:R11.

15. Sardiello M, Licciulli F, Catalano D, Attimonelli M, Caggese C. MitoDrome: a database of *Drosophila melanogaster* nuclear genes encoding proteins targeted to the mitochondrion. Nucleic Acids Res. 2003;31:322–4.

16. Ai W-M, Chen S-B, Chen X, Shen X-J, Shen Y-Y. Parallel evolution of IDH2 gene in cetaceans, primates and bats. FEBS Lett. 2014;588:450–4.

17. Mitterboeck TF, Adamowicz SJ. Flight loss linked to faster molecular evolution in insects. Proc R Soc B. 2013;280:20131128.

18. Yang Y, Xu S, Xu J, Guo Y, Yang G. Adaptive evolution of mitochondrial energy metabolism genes associated with increased energy demand in flying insects. PLoS One. 2014;9:e99120.

19. Brook WJ, Diaz-Benjumea FJ, Cohen SM. Organizing spatial pattern in limb development. Annu Rev Cell Dev Biol. 1996;12:161–80.

20. Paul L, Wang S-H, Manivannan SN, Bonanno L, Lewis S, Austin CL, et al. Dpp-induced Egfr signaling triggers postembryonic wing development in *Drosophila*. Proc Natl Acad Sci USA. 2013;110:5058–63.

21. Yang X, Liu X, Xu X, Li Z, Li Y, Song D, et al. Gene expression profiling in winged and wingless cotton aphids, *Aphis gossypii* (Hemiptera: Aphididae). Int J Biol Sci. 2014;10:257–67.

22. Brisson JA, Davis GK, Stern DL. Common genome-wide patterns of transcript accumulation underlying the wing polyphenism and polymorphism in the pea aphid (*Acyrthosiphon pisum*). Evol Dev. 2007;9:338–46.

23. Xue J, Zhang XQ, Xu HJ, Fan HW, Huang HJ, Ma XF, et al. Molecular characterization of the flightin gene in the wing-dimorphic planthopper, *Nilaparvata lugens*, and its evolution in Pancrustacea. Insect Biochem Mol Biol. 2013;43:433–43.

24. Vigoreaux JO, Hernandez C, Moore J, Ayer G, Maughan D. A genetic deficiency that spans the flightin gene of *Drosophila melanogaster* affects the ultrastructure and function of the flight muscles. J Exp Biol. 1998;201:2033–44.

25. Kvist J, Mattila ALK, Somervuo P, Ahola V, Koskinen P, Paulin L, et al. Flight-induced changes in gene expression in the Glanville fritillary butterfly. Mol Ecol. 2015;24:4886–900.

26. Jones CM, Papanicolaou A, Mironidis GK, Vontas J, Yang Y, Lim KS, et al. Genomewide transcriptional signatures of migratory flight activity in a globally invasive insect pest. Mol Ecol. 2015;24:4901–11.

27. Wojtas K, Slepecky N, Kalm LV, Sullivan D. Flight muscle function in *Drosophila* requires colocalization of glycolytic enzymes. Mol Biol Cell. 1997;8:1665–75.

28. Marden JH, Fitzhugh GH, Girgenrath M, Wolf MR, Girgenrath S. Alternative splicing, muscle contraction and intraspecific variation: associations between troponin T transcripts, Ca2+ sensitivity and the force and power output of dragonfly flight muscles during oscillatory contraction. J Exp Biol. 2001;204:3457–70.

29. Foote AD, Liu Y, Thomas GWC, Vinař T, Alföldi J, Deng J, et al. Convergent evolution of the genomes of marine mammals. Nat Genet. 2015;47:272–5.

30. Mayer C, Sann M, Donath A, Meixner M, Podsiadlowski L, Peters RS, et al. BaitFisher: a software package for multispecies target DNA enrichment probe design. Mol Biol Evol. 2016;33:1875–86.

31. Mitterboeck TF, Liu S, Adamowicz SJ, Fu J, Zhang R, Song W, et al. Supporting data for “Positive and relaxed selection associated with flight evolution and loss in insect transcriptomes”. GigaScience Database 2017. http://dx.doi.org/10.5524/100334

32. Sievers F, Wilm A, Dineen D, Gibson TJ, Karplus K, Li W, et al. Fast, scalable generation of high-quality protein multiple sequence alignments using Clustal Omega. Mol Syst Biol. 2011;7:539.

33. Suyama M, Torrents D, Bork P. PAL2NAL: Robust conversion of protein sequence alignments into the corresponding codon alignments. Nucleic Acids Res. 2006;34:609–12.

34. Penn O, Privman E, Ashkenazy H, Landan G, Graur D, Pupko T. GUIDANCE: A web server for assessing alignment confidence scores. Nucleic Acids Res. 2010;38:23–8. 28.

35. Meredith RW, Janec JE, Gatesy J, Ryder OA, Fisher CA, Teeling EC, et al. Impacts of the Cretaceous terrestrial revolution and KPg extinction on mammal diversification. Science. 2011;334:521–4.

36. Strohm JHT, Gwiazdowski RA, Hanner R. Fast fish face fewer mitochondrial mutations: patterns of dN/dS across fish mitogenomes. Gene. 2015;572:27–34.

37. Castellana S, Vicario S, Saccone C. Evolutionary patterns of the mitochondrial genome in Metazoa: exploring the role of mutation and selection in mitochondrial protein–coding genes. Genome Biol Evol. 2011;3:1067–79.

38. Pons J, Ribera I, Bertranpetit J, Balke M. Nucleotide substitution rates for the full set of mitochondrial protein-coding genes in Coleoptera. Mol Phylogenet Evol. 2010;56:796–807.

39. Foottit RJ, Adler PH. Insect Biodiversity: Science and Society. Chichester, UK: John Wiley & Sons; 2009.

40. Eo SH, Dewoody JA. Evolutionary rates of mitochondrial genomes correspond to diversification rates and to contemporary species richness in birds and reptiles. Proc R Soc B. 2010;277:3587–92.

41. Resh VH, Carde RT, editors. Encyclopedia of Insects, 2nd Edition. Elsevier; 2009.

42. Evans PD, Wigglesworth VB. Advances in insect physiology. Florida, USA.: Academic Press Inc.; 1988.

43. Conley KE, Lindstedt SL. Energy-saving mechanisms in muscle: the minimization strategy. J Exp Biol. 2002;205:2175–81.

44. McMahon DP, Hayward A, Kathirithamby J. The first molecular phylogeny of Strepsiptera (Insecta) reveals an early burst of molecular evolution correlated with the transition to endoparasitism. PLoS One. 2011;6:e21206.

45. Gregory TR. Genome size and developmental complexity. Genetica. 2002;115:131–46.

46. Pavlidis P, Jensen JD, Stephan W, Stamatakis A. A critical assessment of storytelling: Gene ontology categories and the importance of validating genomic scans. Mol Biol Evol. 2012;29:3237–48.

47. Shang F, Ding B, Xiong Y, Dou W, Wei D, Jiang H, et al. Differential expression of genes in the alate and apterous morphs of the brown citrus aphid, *Toxoptera citricida*. Nat Sci Reports. 2016;6:32099.

48. Xue J, Bao Y-Y, Li B-L, Cheng Y-B, Peng Z-Y, Liu H, et al. Transcriptome analysis of the brown planthopper *Nilaparvata lugens*. PLoS One. 2010;5:e14233.

49. Keren H, Lev-Maor G, Ast G. Alternative splicing and evolution: diversification, exon definition and function. Nat Rev Genet. 2010;11:345–55.

50. Marden JH. Functional and ecological effects of isoform variation in insect flight muscle. In: Vigoreaux JO, editor. Nature's Versatile Engine Insect Flight Muscle Inside and Out. New York: Springer Science+Business Media; 2006. p. 214–29.

51. Marden JH, Fescemyer HW, Saastamoinen M, Macfarland SP, Vera JC, Frilander MJ, et al. Weight and nutrition affect pre-mRNA splicing of a muscle gene associated with performance, energetics and life history. J Exp Biol. 2008;211:3653–60.

52. Pan Q, Shai O, Lee LJ, Frey BJ, Blencowe BJ. Deep surveying of alternative splicing complexity in the human transcriptome by high-throughput sequencing. Nat Genet. 2008;40:1413–6.

53. Waveren C Van, Moraes CT. Transcriptional co-expression and co-regulation of genes coding for components of the oxidative phosphorylation system. BMC Genomics. 2008;9.

54. Deak BII, Bellamy PR, Bienz M, Dubuis Y, Fenner E, Gollin M, Rahmi A, Ramp T, Reinhardt CA, Cotton B. Mutations affecting the indirect flight muscles of *Drosophila melanogaster*. J Embryol Exp Morphol. 1982;69:61–81.

55. Mallick S, Gnerre S, Muller P, Reich D. The difficulty of avoiding false positives in genome scans for natural selection. Genome Res. 2009;19:922–33.

56. Gharib WH, Robinson-Rechavi M. The branch-site test of positive selection is surprisingly robust but lacks power under synonymous substitution saturation and variation in GC. Mol Biol Evol. 2013;30:1675–86.

57. Sun Y-B, Zhou W-P, Liu E-Q, Irwin DM, Shen Y-Y, Zhang Y-P. Genome-wide scans for candidate genes involved in the aquatic adaptation of dolphins. Genome Biol Evol. 2012;5:130–9.

58. Faria D, Schlicker A, Pesquita C, Bastos H, Ferreira AEN, Albrecht M, et al. Mining GO annotations for improving annotation consistency. PLoS One. 2012;7:e40519.

59. Hahn MW, Han M V, Han S-G. Gene family evolution across 12 *Drosophila* genomes. PLoS Genet. 2007;3:e197.

60. De Grassi A, Lanave C, Saccone C. Genome duplication and gene-family evolution: the case of three OXPHOS gene families. Gene. 2008;421:1–6.

61. Petersen M, Meusemann K, Donath A, Dowling D, Liu S, Peters SR, et al. Orthograph: a versatile tool for mapping coding nucleotide sequences to clusters of orthologous genes. BMC Bioinformatics. 2017;18:111.

62. Katoh K, Standley DM. MAFFT multiple sequence alignment software version 7: improvements in performance and usability. Mol Biol Evol. 2013;30:772–80.

63. Zhang J, Nielsen R, Yang Z. Evaluation of an improved branch-site likelihood method for detecting positive selection at the molecular level. Mol Biol Evol. 2005;22:2472–9.

64. Yang Z. PAML 4: phylogenetic analysis by maximum likelihood. Mol Biol Evol. 2007;24:1586–91.

65. Yang Z. Likelihood ratio tests for detecting positive selection and application to primate lysozyme evolution. Mol Biol Evol. 1998;15:568–73.

66. Benjamini Y, Hochberg Y. Controlling the false discovery rate: a practical and powerful approach to multiple testing. J R Stat Soc Ser B. 1995;57:289–300.

67. Stone G, French V. Evolution: Have wings come, gone and come again? Curr Biol. 2003;13:R436–8.

68. Hughes AL. Looking for Darwin in all the wrong places: the misguided quest for positive selection at the nucleotide sequence level. Heredity. 2007;99:364–73.

69. Huang DW, Sherman BT, Lempicki RA. Bioinformatics enrichment tools: paths toward the comprehensive functional analysis of large gene lists. Nucleic Acids Res. 2009;37:1–13.

70. Huang DW, Sherman BT, Lempicki RA. Systematic and integrative analysis of large gene lists using DAVID bioinformatics resources. Nat Protoc. 2009;4:44–57.

71. Mi H, Poudel S, Muruganujan A, Casagrande JT, Thomas PD. PANTHER version 10: expanded protein families and functions, and analysis tools. Nucleic Acids Res. 2016;44:D336-42.

72. Lewin A, Grieve IC. Grouping Gene Ontology terms to improve the assessment of gene set enrichment in microarray data. BMC Bioinformatics. 2006;7.

73. R Core Team. R: A language and environment for statistical computing. R Found. Stat. Comput. Vienna, Austria. URL http//www.R-project.org/. 2013.

74. Pond SLK, Frost SDW, Muse SV. HyPhy: hypothesis testing using phylogenies. Bioinformatics. 2005;21:676–9.

75. Pond SLK, Murrell B, Fourment M, Frost SDW, Delport W, Scheffler K. A random effects branch-site model for detecting episodic diversifying selection. Mol Biol Evol. 2011;28:3033–43.

76. Delport W, Poon AFY, Frost SDW, Pond SLK. Datamonkey 2010: A suite of phylogenetic analysis tools for evolutionary biology. Bioinformatics. 2010;26:2455–7.

FIGURE LEGENDS

Figure 1. Tree topology and species used in analyses of nuclear genes. Species names followed by a star indicate those species used in positive selection analysis associated with the origin of Pterygota (branch ‘P’) and other lineages for comparison (branches ‘U’ and ‘D’). Circles or squares on the branches indicate each of the 11 lineages that were used in positive selection analysis of flight loss, with circles indicating full flight loss and squares indicating female-only

flight loss. Triangles indicate related flight-capable branches used for comparison with the lineages representing a loss of flight in positive selection analysis. Note that sub-trees were used for the positive selection tests, and so not all species shown here were included. The colour of the circles or squares indicates the estimated degree of accuracy in the phylogenetic mapping of the flight loss, given the available taxonomic sampling (green = good, orange = fair, grey = approximate). Red lineages (fully flightless) were compared with blue lineages (related flying) in the nuclear gene analyses of relaxed selection (dN/dS ratios) associated with flightlessness, with all other lineages used for a background rate. A similar (smaller) tree was used for mitochondrial gene analyses of relaxed selection where both red (fully flightless) and purple (female-only flightless) lineages were compared with blue (related flying) lineages, with other lineages representing the background rate.

Figure 2. dN/dS ratios in flightless vs. related flying lineages for 13 mitochondrial protein-coding genes. In 11 of 13 genes, the dN/dS ratio in the flightless pterygote lineages is higher than the dN/dS ratio of flying lineages. Genes with a significant difference in rates (after Bonferroni-Hochberg correction) are marked with '*'; in all five cases, the dN/dS ratio is higher in the flightless lineages than in their flight-capable counterparts. Dashed lines signify the mean dN/dS values; flightless: 0.031, and flying: 0.021. The tree with lineages tested is provided in [31] S10 tree file.

Figure 3. Positive selection in hexapod lineages in nuclear and mitochondrial genes of interest. The tree is adopted from Misof et al. [2], showing orders, and involving one species representative per insect order for each gene tested. Orders/lineages that are shaded grey are

apterygote (i.e. hexapods that never evolved the ability to fly) and those shaded orange consist entirely of species that are flightless due to a secondary loss of flight after its origin in Pterygota; note Embioptera and Strepsiptera are female flightless only. The lineage marked with ‘P’ represents the lineage leading to the clade Pterygota; ‘L’ = polyneoptera, ‘H’ = holometabola (i.e. complete metamorphosis) insects.

Figure1

Click here to download Figure Figure1_png.png

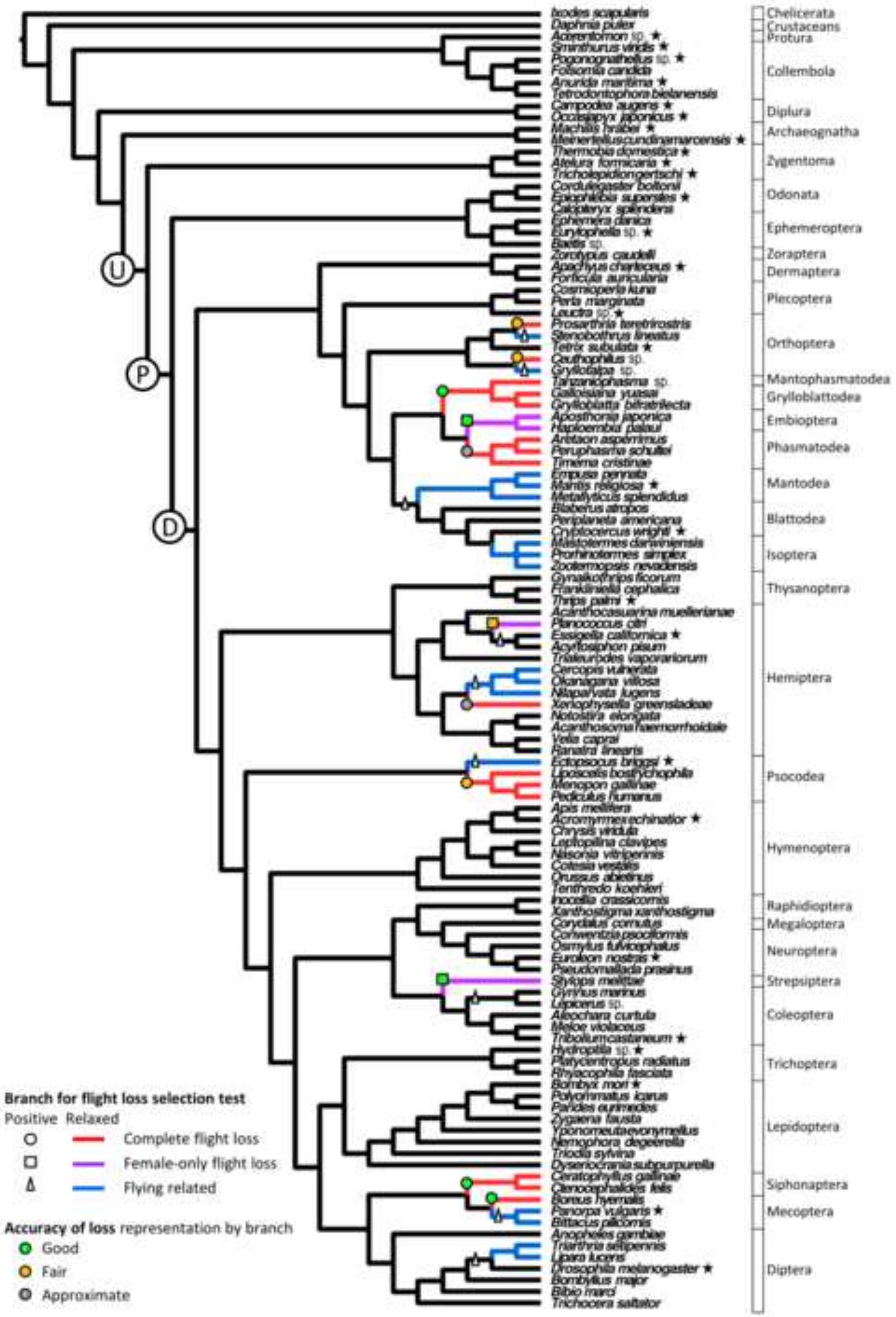

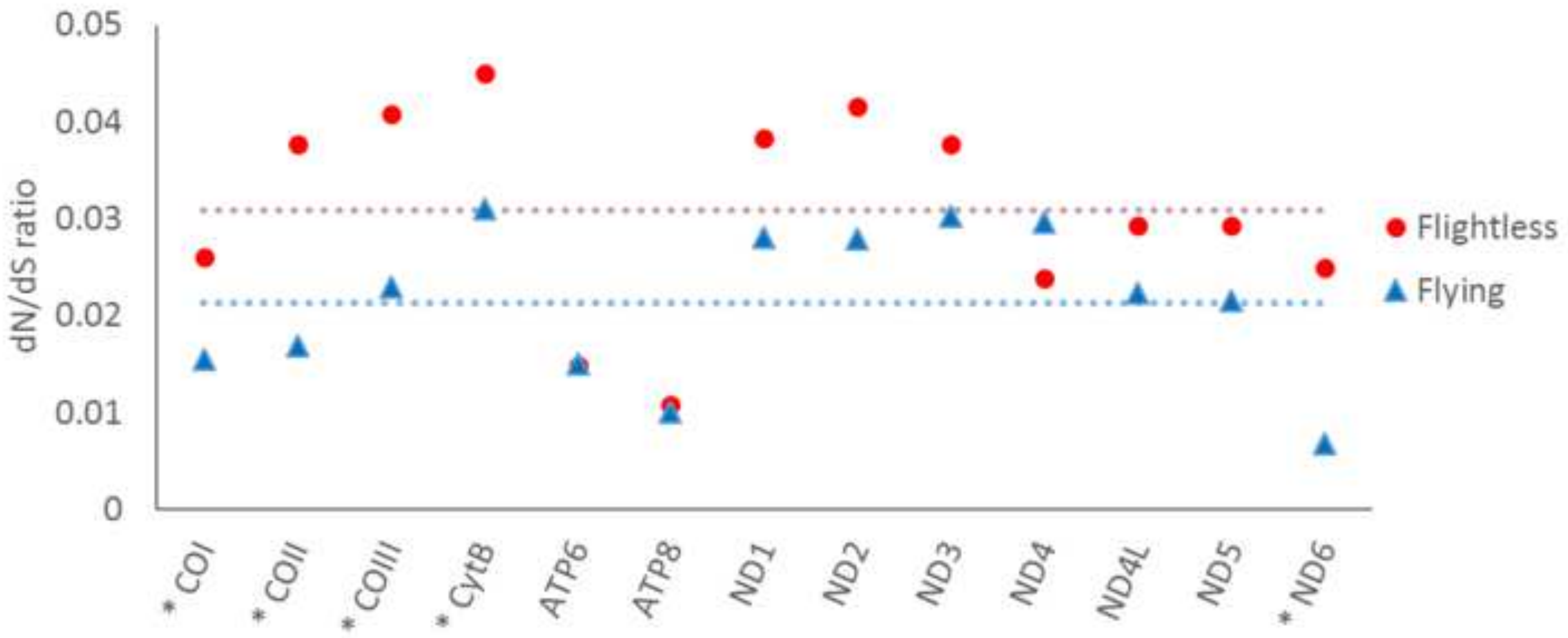
0.05
0.04
0.03
0.02
0.01
0
dN/dS ratio
Flightless
Flying
* COI
* COII
* COIII
* CytB
ATP6
ATP8
ND1
ND2
ND3
ND4
ND4L
ND5
* ND6

Figure3

Click here to download Figure Figure3_png.png

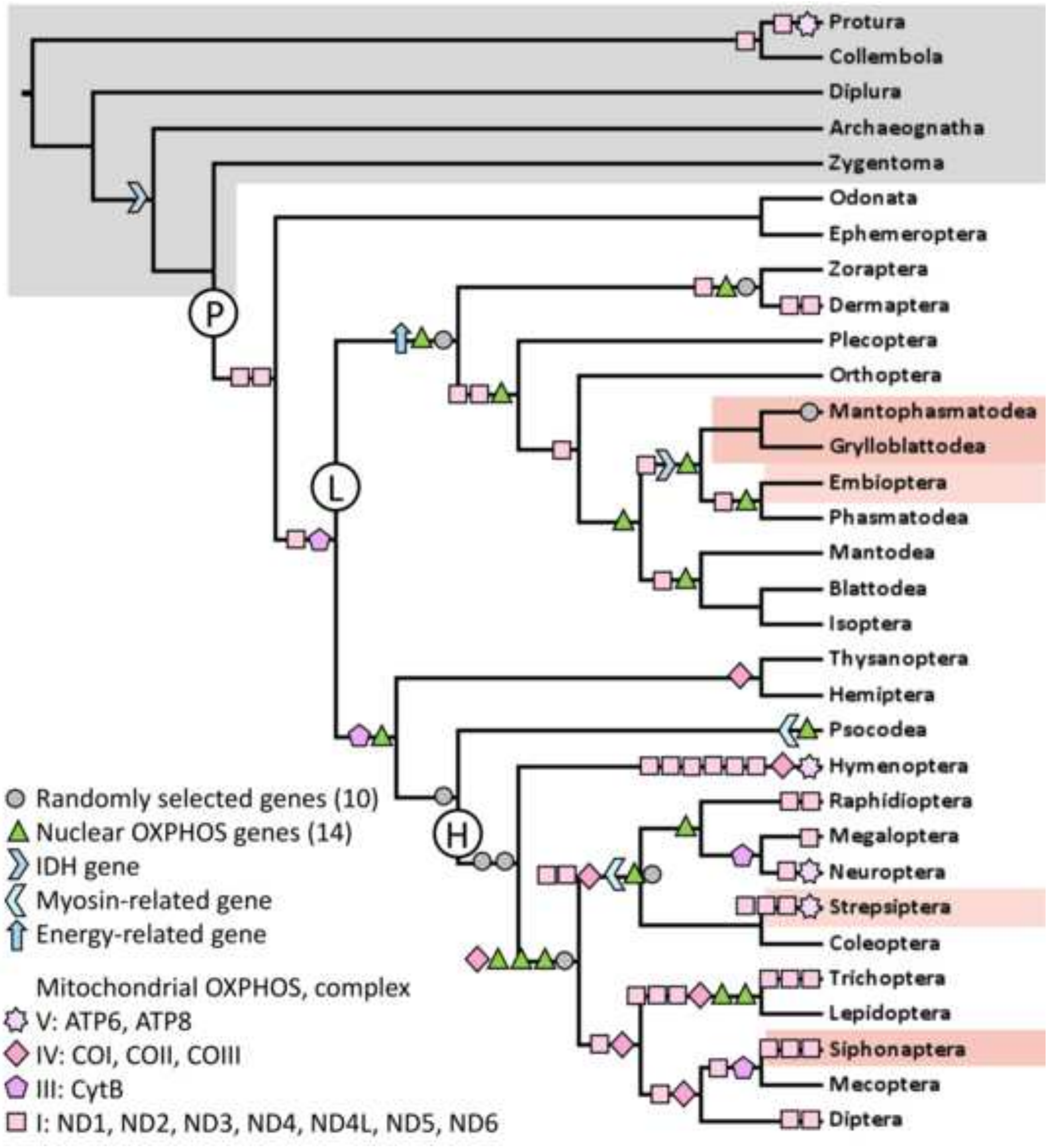

 Click here to download Personal Cover Response Letter_July14,2017.doc

Response Letter

July 14th 2017

Manuscript # GIGA-D-17-00053

Dear Dr. Hans Zauner, Editor, GigaScience

Please find submitted the manuscript entitled “Positive and relaxed selection associated with flight evolution and loss in insect transcriptomes”, which was deemed potentially acceptable for publication pending minor revisions.

We are glad both reviewers found the manuscript well-written and the methods thorough. We have made the suggested minor changes to the manuscript, outlined further below. The main changes were to add sections of interpretation to the discussion, as suggested by the reviewers, and to provide more detail on the genes of interest. We think the changes have improved the manuscript.

We thank the reviewers and editor for their thoughtful input and hope that you will now find the manuscript acceptable for publication in *GigaScience*. We look forward to hearing back from you soon.

Yours sincerely,

Fatima Mitterboeck

____

Dear Dr. Mitterboeck,

Your manuscript "Positive and relaxed selection associated with flight evolution and loss in insect transcriptomes" (GIGA-D-17-00053) has been assessed by our reviewers. Based on these reports, and my own assessment as Editor, I am pleased to inform you that it is potentially acceptable for publication in GigaScience, once you have carried out some essential revisions suggested by our reviewers.

The reviewers have no substantial concerns regarding the overall methodology,however, they do point out that the conclusions that can be drawn from your work are rather limited - but we agree that the work is nevertheless a useful contribution and suitable for publication in GigaScience, in principle. Please revise your paper in light of the reviewers recommendations, in particular regarding caveats and limitations of your work mentioned by the referees.

Their reports, together with any other comments, are below. Please also take a moment to check our website at http://giga.edmgr.com/ for any additional comments that were saved as attachments.

If you prepare your revised manuscript, please amend your reference list with a citation to any data that

will be published via our GigaDB server, and cite this dataset in your data availability section and elsewhere in the manuscript, where appropriate.

The format should be like this (with your own author list and paper title, of course):

[xx] Zheng L-Y, Guo X-S, He B, Sun L-J, Peng Y, Dong S-S, et al. Supporting data for " Genome data from sweet and grain sorghum (Sorghum bicolor)". GigaScience Database. 2017. http://dx.doi.org/XXXX

Prior to publication, we will update the citation with the actual doi of the dataset.

Response:

We have added the reference to the dataset as an in-text citation [31] and to the reference list as:

Mitterboeck TF, Liu S, Adamowicz SJ, Fu J, Zhang R, Song W, et al. Supporting data for "Positive and relaxed selection associated with flight evolution and loss in insect transcriptomes". GigaScience Database 2017. http://dx.doi.org/XXXX

We have replaced all mention of Additional File 1 (AF1) in the manuscript text with the citation [31] to this data set.

Once you have made the necessary corrections, please submit a revised manuscript online at:

http://giga.edmgr.com/

If you have forgotten your username or password please use the "Send Login Details" link to get your login information. For security reasons, your password will be reset.

Please include a point-by-point within the 'Response to Reviewers' box in the submission system. Please ensure you describe additional experiments that were carried out and include a detailed rebuttal of any criticisms or requested revisions that you disagreed with. Please also ensure that your revised manuscript conforms to the journal style, which can be found in the Instructions for Authors on the journal homepage.

The due date for submitting the revised version of your article is 10 Aug 2017.

We look forward to receiving your revised manuscript soon.

Best wishes,

Hans Zauner
GigaScience
www.gigasciencejournal.com

Reviewer reports:

Reviewer #1: This is a fairly concisely and effectively written report of a large comparative genomics study. The hypothesis is that the evolutionary transition to flight, and subsequent independent losses of flight, in pterygote insects involved detectable changes in selective pressures and adaptation in a large set of single-copy, orthologous nuclear and mitochondrial genes. The results are not as clear-cut as similar studies have shown for birds and especially bats, but the authors do a nice job discussing caveats, particularly the much longer evolutionary time involved in insect divergences, which serves to obscure the signal. Figure 2 contains what I think is the most noteworthy result, and which is very well discussed in L 265-279.

General remarks:

The methods are at the end, so it would be helpful to prepare the reader in the introductory parts regarding the distinction between positive selection and relaxation of purifying selection. Both are detected by an increase in dN/dS, but positive selection requires the ratio to exceed 1.0 (correct? If not, explain). Some introductory discussion of the difference between positive and relaxed selection would enrich the paper and better engage the reader in the results.

Response:

We have added to the beginning of the first results section, at line 156:

"Tests of positive selection were performed for each lineage of interest via branch-site models, which estimate dN/dS ratios at each codon site and between branches such that positive selection is detected in the lineage of interest if a subset of codon sites have dN/dS ratios greater than 1, while the other lineages have ratios less than 1 or equal to 1, indicating purifying selection or neutral evolution, respectively."

We have also added at the beginning of the relaxed selection results, at line 217:

"Postulated relaxed selection was detected by increased dN/dS ratios across the fully-flightless vs. flight-capable branches of the tree (i.e. pooling branches by flight state) calculated for the entire length of each gene tested, as opposed to positive selection which was detected using branch-site models (accounting for dN/dS ratios at each site) on individual lineages of interest."

This sets up how the analyses were conducted and the main differences between positive and relaxed selection analysis, which are 1) dN/dS at sites vs. whole gene length; and 2) individual lineage tested vs. multiple lineages tested together.

We have also added some detail on what the results for functional tests represent in the results section, to allow greater understanding for the reader. In the Table 1 caption, at line 185, we have added:

"Statistical over-representation is tested by modified Fisher's exact tests in DAVID and binomial statistics in PANTHER, with raw p values provided here."

There is only one sentence in this paper (L 390) explaining that changes in gene regulation are important for adaptation. The next sentence mentions the need for future studies of this nature to include non-coding regions, but places this in the context of gene gains and losses. I think there needs to be a clear and unequivocal statement that this study examines only coding regions and does not consider changes in gene regulatory regions that affect things like gene co-regulation, which are demonstrably important in e.g. energy metabolism. Doing this will help set up the finding about genes involved in the regulation of alternative splicing, which may be the tip of the icebeaker for broader regulatory changes.

Response: We have added the following sentences after the discussion of splicing genes, at Line 406:

"This study examined only coding regions and was not able to consider changes in gene regulatory regions, which affect co-regulation. Co-regulation is important in processes including energy production [53]. Our results that suggest alternative splicing is an important gene functional category for flight evolution may be a symptom of the involvement of regulatory changes in general, which we were not able to test here."

53. Waveren C Van, Moraes CT. Transcriptional co-expression and co-regulation of genes coding for components of the oxidative phosphorylation system. BMC Genomics. 2008;9.

It might be worth discussing how/why a gene would show signals of positive selection with flight loss. That would mean that in multiple loss-of-flight lineages, the same ortholog adaptively diverged from flight-adapted forms. This would require, I think, that specific amino acids within proteins are required for flight. Discussing this in a bit of detail would help both the authors and readers see why this is a rather exotic hypothesis, especially after hundreds of millions of years of flight cabability (and hence no clear path back the way they came). Flight requires high capacity: lots of mitochondria packed with membranes and hugely more oxygen supply than required during rest. Metabolic flux and oxygen conductivity matter enormously. Are these things in any system known to be controlled by particular amino acid substitutions in particular genes? I can't readily think of any examples. I do know of a paper that shows that one alternative splice form of one metabolic gene is required for high metabolic flux in flight muscles: Mol. Biol. Cell 8, 1665-1675.

Response: We have added the suggestion about how a gene would show signal of positive selection, at line 426:

“For a gene to show signals of positive selection in multiple flight loss examples, the same ortholog must have adaptively diverged from flight-capable lineages. Given the long evolutionary history in the flight-adapted state before flight losses occurred and the seeming ease in which flight ability can be ‘turned off’ developmentally by loss of function of specific genes [54], the relaxed selection tests may be more able to uncover trends in genes associated with flight loss than positive selection. Even so, given the consistent association between flight loss and increased reproductive ability, future studies using more genomic information may uncover positive selection with flight loss that we did not, or were not able to, detect here.”

We thank the reviewer for the insightful suggestion. We could not think of any further biological interpretation than previously given (lines 422-426, reproduction, sugar metabolism) for the expected functional categories associated with the positive selection analysis of flight loss, and we did not want to risk over-interpreting our results.

The underlying idea in this study is that flight capability is the major selective factor for flighted vs. unflighted insects. I don't dispute that, but it is worth mentioning that a flight-to-flightless transition will likely involve major changes in ecology and life history, which could include diet, predators, dispersal, phenology, courtship, and so forth. These factors are likely to be species-specific. How would this affect the present results? (I noticed belatedly that the paper discusses this briefly in terms of expecting positive selection in loss of flight lineages arising from dispersal-reproduction tradeoffs. I think you could do more in this regard.)

Response: We have added at line 412:
“The loss of flight is not only associated with the change in flight ability, but also major changes in ecology and life history, such as diet, predation, habitat (e.g. woodlands, deserts), courtship, and often reduction in dispersal ability [8][17]. Such changes are specific to certain species or clades, and thus the use of multiple lineages may help to eliminate some noise created by confounding biological or ecological factors. Nonetheless, some associated factors, such as reduced dispersal ability, are likely commonly associated with flight loss, and therefore, the results here are likely impacted by co-occurring factors in addition to change in flight capability itself.”

Specific remarks:
L73. 406 MYA grossly overstates the precision of what we know about this date. You should say something like "approximately 400 MYA".

Response: 406 has been changed to 400 in this sentence (line 73) and also at line 310, both of which now read “approximately 400 million years ago”.

L.75. There is a more recent paper that is the more appropriate citation because it addresses more basal insects: PNAS 112 (52), 15946-15951 (2015).

Response: Thank you for the suggestion. We have added this citation (line 75). We opted to keep the other citations as well to show the diversity of hypotheses on insect flight origin.

L78. "at least incomplete metamorphosis". This is awkward wording and difficult to understand until reaching the later part of the sentence. Rewrite this sentence.

Response: This sentence has been reworded as (line 77):

"In addition to the evolution of flight, pterygote insects evolved incomplete metamorphosis, which involves egg, nymph, and adult stages."

Based on this change we also added "within Pterygota" in the following sentence (line 79) to clarify that other metamorphosis types occur in pterygotes in addition to incomplete metamorphosis.

L100-106. Here it you should also state that the the particular splice form of certain genes has been shown to be necessary for insect flight (e.g. Mol. Biol. Cell 8, 1665-1675) and that the relative abundance of different splice variants affects the power output of flight muscle in a basal pterygote (Journal of Experimental Biology 204 (20), 3457-3470). Doing so may help explain the result, although without any genes being named or shown in a table (consider doing so, it's only 39 genes) I can't tell if any of these are known to be related to anything related to metabolism, flight, or life history.

Response: we have added this information at line 112, along with the two references:

"Additionally, particular splice forms of certain genes such as encoding glycerol-3-phosphate dehydrogenase (functions in the glycolytic pathway to produce ATP) appear necessary for flight [27], with the relative abundance of various splice variants affecting the power output of flight muscles, as shown in a dragonfly species [28]."

27. Wojtas K, Slepecky N, Laurence VK, Sullivan D. Flight muscle function in *Drosophila* requires colocalization of glycolytic enzymes. Mol Biol Cell. 1997;8:1665–75.

28. Marden JH, Fitzhugh GH, Girgenrath M, Wolf MR, Girgenrath S. Alternative splicing, muscle contraction and intraspecific variation: associations between troponin T transcripts, Ca2+ sensitivity and the force and power output of dragonfly flight muscles during oscillatory contraction. J Exp Biol. 2001;204:3457–70.

We have added some candidate gene names to the text in each results section, where the genes relate to wing or mitochondrial functions. Often, genes do not have descriptive names, so we opted to provide some examples in the main prose, rather than the whole candidate gene lists, which would be long given the multiple tests.

Additionally, we have now provided the list of all gene names and descriptions in the Supplementary Material Table S15, with information columns to be able to sort the genes by analysis (e.g. candidate, background, analysis 1, 2, 3). As well, we have added the DAVID gene ontology category results in the Supplementary Material Table S16, in case of database changes.

In conjunction with the other reviewer's suggestion to add more detailed gene category information, we have created some categories of genes of potential interest by grouping together similar GO terms, 1) related to wing development, and 2) related to mitochondrion, ATP-binding, and the electron transport chain, etc. Grouping of GO terms is suggested in the literature to improve detection of trends since multiple terms overlap in their gene lists. The individual GO categories of interest do not show up the candidate gene lists from the DAVID analysis, unless they are over-represented. This way, we have performed the statistical tests ourselves and reported the results for those particular categories of interest. However, there were no significant over- or under-representation of these gene categories in the candidate gene lists, and so the interpretation was not affected by these additions.

The new sections with the gene names and results for grouped functional terms are at lines 165-174 (analysis 1), lines 199-203 (analysis 2), and lines 226-230 (analysis 3).

L 112-113: "... during the time when flight originated, during a time span of approximately 14 million years [2]." This is similar to my remark about L73 (above). 14MYA is an estimate from a molecular evolution study, and there is very scant (arguably no) fossil evidence for the time of origin of pterygotes. What do you think the error bounds are in this 14 MYA number? I'd guess at least 40 MY and possibly much more.

Response: We have removed the phrase "during a time span of approximately 14 million years" from line 123. We have also removed a similar time estimate from molecular study from line 532 "each representing a time span of an estimated 20 million years".

342 " ... energy levels needed for multiple flight performances". Not clear what this means. I think you mean to say "allow quantitative adjustment of muscle force and power output". Another meaningful reference here is: Journal of Experimental Biology 211 (23), 3653-3660.

Response: We have changed the phrasing to the reviewer's suggested phrasing and have added the suggested reference to the sentence, at line 393:

"Alternative splicing allows various isoforms of muscle-related molecules and as such appears to be an important mechanism to allow quantitative adjustment of muscle force and power output [50][51]"

51. Marden JH, Fescemyer HW, Saastamoinen M, Macfarland SP, Vera JC, Frilander MJ, et al. Weight and nutrition affect pre-mRNA splicing of a muscle gene associated with performance, energetics and life history. J Exp Biol. 2008;211:3653–60.

L. 348: I don't understand this sentence. What does "flight morphs within species" mean in the context of this study? How does this sentence relate to the next sentence?

Response: the two sentences have been rephrased for clarity, at line 401-404, as:

“However, flightless vs. flying morphs of insects do not represent evolutionarily distinct lineages, and so genes exhibiting different expression levels among morphs may not be those bearing signatures of differences in selection regime between flightless vs. flying insects on much longer evolutionary timescales. Thus, we suggest genes related to splicing are a potential category for further investigation of whether differing selection pressures occurred with the origination of flight and flight loss in insects.”

Reviewer #2: The authors use data from the 1KITE project and additional genomic data from Misof et al. (2014, Science) to infer positive/relaxed selection on a selection of over 1000 nuclear and mitochondrial genes that are potentially involved in the loss/gain of insect flight.

Overall, I thought the manuscript was well written and the analyses very thorough albeit with limited conclusions. The authors use a comprehensive sequence data set from diverse insect fauna and try to match the phylogeny of flight gain/loss with models of dN/dS as measures of selection. In my opinion the main conclusions are (i) the evidence for relaxed selection in mtOXPHOS genes mirroring that of other taxa and (ii) the association of 'splicing' with flight loss although it would have been nice to see some extension work to the latter finding rather than simple speculation in the Discussion.

As I say I don't think the results are ground-breaking but I did appreciate the authors' efforts to spell out the caveats with their approach and in doing so they mainly addressed my comments as I was reading the paper. For example, I would be keen to see the data for more specialised gene categories.

Response: We have added analyses examining the proportion of genes in the candidate and non-candidate gene lists that fit into potentially relevant gene categories. We created categories of genes of potential interest by grouping together similar GO terms, 1) related to wing development, and 2) related to mitochondrion, ATP-binding, and the electron transport chain, etc. Grouping of GO terms is suggested in the literature, as it can increase the ability to detect trends when GO terms are overlapping. The individual GO categories of interest do not show up the candidate gene lists from the DAVID analysis, unless they are over-represented. We report the results of these larger categories in the manuscript; however, since the genes in the candidate gene lists do not significant over- or under-represent these categories, the interpretation is not changed.

Additionally, we have now provided the list of all gene names and descriptions in the Supplementary Material Table S15, with information columns to be able to sort the genes by analysis (e.g. candidate, background, analysis 1, 2, 3). As well, we have added the DAVID gene ontology category results in the Supplementary Material Table S16, in case of database changes.

Furthermore (in response to a further comment below), we have added the gene names and their joint functional category for the two genes which were overlapping between the flight gain (positive

selection) and flight loss (relaxed selection) analyses, as those two genes were the most likely to be of importance (at lines 253-260).

While the other reviewer suggested adding gene lists into the manuscript, many of the genes do not have descriptive names. So, in the main prose, we opted to report only some examples of genes in the candidate lists, where they fall into these categories of interest.

The new sections with the gene names and results for grouped functional terms are at lines 165-174 (analysis 1), lines 199-203 (analysis 2), and lines 226-230 (analysis 3).

I was also curious as to how you determine whether the signatures of selection are truly related to flight and not some pleiotropic phenotype (e.g. delayed reproduction). But again, these concerns were at least highlighted in the Discussion.

Response: the other reviewer had also suggested more discussion on these potential confounding factors and so we elaborated slightly on this point, at line 412-419:

"The loss of flight is not only associated with the change in flight ability, but also major changes in ecology and life history, such as diet, predation, habitat (e.g. woodlands, deserts), courtship, and often reduction in dispersal ability [8][17]. Such changes are specific to certain species or clades, and thus the use of multiple lineages may help to eliminate some noise created by confounding biological or ecological factors. Nonetheless, some associated factors, such as reduced dispersal ability, are likely commonly associated with flight loss, and therefore, the results here are likely impacted by co-occurring factors in addition to change in flight capability itself."

Major comments

I see the benefits of the GO-term approach but is this a bit simplistic? Especially when you are only sampling from a relatively small number of genes (n = ~1000). I was wondering whether there is any way to correlate dN/dS with the timing of flight gain/loss from your phylogenetic records - would any correlation in say, one of the genes showing relaxed selection associated with flight loss, provide useful additional information?

Response: We thank the reviewer for this interesting idea. We understood the suggestion to mean to consider testing whether more ancient cases of flight loss show higher dN/dS ratios than more recent cases of flight loss for particular genes, and that these genes may be of interest. We have considered the suggestion, but did not think it feasible with the current state of the data. Based on the reviewers' other suggestions about the imprecision of timing events with molecular phylogenies, as well as our other mentioned caveat about the sampling of lineages making it difficult to pinpoint the origin of some cases of these flight loss on the phylogeny, we were not confident that those results would prove accurate. This is certainly an approach to consider for future work involving trees with higher sampling of lineages that would have more accurate divergence times between flightless lineages and closest flight-capable sister lineages.

However, we did provide more specific information at the level of genes, as opposed to only examining overlap in the broader GO categories. We have now reported which genes overlap between those detected under positive selection with flight gain and those detected under relaxed selection with flight loss, as we expect those genes showing mirror-image similarities between the two transition directions to be the most relevant (at lines 253-260).

Minor comments:
Line 57 – proteases

Response: This has been changed to "proteases" (line 57)

Line 71 - 'likely positively' is not correct terminology

Response: This phrase has been changed to "most likely had a positive impact on" (line 71)

Line 82 - 'an estimated thousand of times' doesn't sound right

Response: we have changed this phrasing to "it has been estimated that flight has been lost thousands of times within pterygotes" (line 82-83)

Background, paragraph 3 - the authors may be interested in differentially expressed candidate gene sets associated with flight activity in the Glanville fritillary butterfly (Kvist et al., 2015, Molecular Ecology) and the cotton bollworm moth, Helicoverpa armigera (Jones et al. 2015, Molecular Ecology) to enhance discussion and comparisons.

Response: We have added one sentence with information from both references at line 108-112:

"Functions of genes observed to be differentially expressed between flying insect individuals with higher vs. lower flight metabolic rate include ribosome/RNA processing [25], while genes exhibiting differences between long- vs. short-distance flight migrators include those involved in lipid mobilization and flight muscle structure [26]."

25. Kvist J, Mattila ALK, Somervuo P, Ahola V, Koskinen P, Paulin L, et al. Flight-induced changes in gene expression in the Glanville fritillary butterfly. Mol Ecol. 2015;24:4886–900.

26. Jones CM, Papanicolaou A, Mironidis GK, Vontas J, Yang Y, Lim KS. Genomewide transcriptional signatures of migratory flight activity in a globally invasive insect pest. Mol Ecol. 2015;24:4901–11.

We have received recommendations for additional references and information from the other reviewer as well, so we chose to keep each addition relatively short, in line with the previous concise descriptions earlier in the paragraph of the introduction.

Line 134 – guidelines

Response: "guideline" has been changed to "guidelines" (line 144)

Line 198 - were significant

Response: We have changed "four of those as significant difference" to "four of those exhibited a significant difference" (line 234)

Line 199 - what does ~ mean in the context of '~0'?

Response: We have replaced "~0" with "$1.0x10^{-16}$" (line 235)

I think for the lay reader at times in the main body of text it would be nice to include an example of a common name species related the insect Order (e.g. Thysanoptera, thrips). It helps the reader and improves the accessibility of the paper.

Response: We have added common names within lines:

Line 63 (abstract): "holometabolous (complete metamorphosis)"

Line 269-271: "The apterygote lineages (i.e. primarily flightless lineages, highlighted in grey) as well as lineages in orders Odonata (i.e. dragonflies and damselflies) and Ephemeroptera (i.e. mayflies),"

Line 277: "holometabolous (i.e. complete metamorphosis) insect clade"

Line 350-351: "Hymenoptera (i.e. bees, wasps, ants, sawflies), Coleoptera (i.e. beetles), Strepsiptera (i.e. twisted-wing parasites), Diptera (i.e. flies), and Thysanoptera (i.e. thrips) [41]."

Other changes:

We have modified a previous phrase in the acknowledgment section to add our thanks to the two reviewers by the names provided in their reviews, at line 677-678:

"and Jim Marden and Christopher Jones for valuable input on the manuscript as reviewers."

In results section 1 (line 174-175) we changed the Fisher's exact test to be for 39 candidate genes that were unique to the Pterygota lineage instead of 126 candidate genes that were detected but not necessarily unique. The change was made in order to fit with the reporting of newly added results for the 39 unique candidate genes.

As an addition in the discussion caveat section, we acknowledge that our sample size of genes is low within some Gene Ontology categories (line 463-465):

"Many gene functional categories are poorly represented in our data set, and thus the "expected" counts are low in some categories. The results of this study might be therefore be considered hypotheses for testing using a larger portion of genome in future studies."

We thank the Editor for the careful review of our paper. We think that addressing these comments has strengthened the quality and readability of the paper for future readers.
