# Supplementary material for: Positive and relaxed selection associated with flight evolution and loss in insect transcriptomes
Source: Gigascience. 2017 Aug 16;6(10):1–14. doi: 10.1093/gigascience/gix073 (PMC5632299; doi:10.1093/gigascience/gix073)
Supplement: Response-to-Reviewer-Comments_Original-Submission.pdf [file gix073_Response-to-Reviewer-Comments_Original-Submission.pdf]

Author's Response To Reviewer Comments

Response Letter
July 14th 2017

Manuscript # GIGA-D-17-00053

Dear Dr. Hans Zauner, Editor, GigaScience

Please find submitted the manuscript entitled “Positive and relaxed selection associated with flight evolution and loss in insect transcriptomes”, which was deemed potentially acceptable for publication pending minor revisions.

We are glad both reviewers found the manuscript well-written and the methods thorough. We have made the suggested minor changes to the manuscript, outlined further below. The main changes were to add sections of interpretation to the discussion, as suggested by the reviewers, and to provide more detail on the genes of interest. We think the changes have improved the manuscript.

We thank the reviewers and editor for their thoughtful input and hope that you will now find the manuscript acceptable for publication in GigaScience. We look forward to hearing back from you soon.

Yours sincerely,
Fatima Mitterboeck

Dear Dr. Mitterboeck,

Your manuscript "Positive and relaxed selection associated with flight evolution and loss in insect transcriptomes" (GIGA-D-17-00053) has been assessed by our reviewers. Based on these reports, and my own assessment as Editor, I am pleased to inform you that it is potentially acceptable for publication in GigaScience, once you have carried out some essential revisions suggested by our reviewers.

The reviewers have no substantial concerns regarding the overall methodology,however, they do point out that the conclusions that can be drawn from your work are rather limited - but we agree that the work is nevertheless a useful contribution and suitable for publication in GigaScience, in principle. Please revise your paper in light of the reviewers recommendations, in particular regarding caveats and limitations of your work mentioned by the referees.

Their reports, together with any other comments, are below. Please also take a moment to check our website at http://giga.edmgr.com/ for any additional comments that were saved as attachments.

If you prepare your revised manuscript, please amend your reference list with a citation to any data that will be published via our GigaDB server, and cite this dataset in your data availability

section and elsewhere in the manuscript, where appropriate.

The format should be like this (with your own author list and paper title, of course):

[xx] Zheng L-Y, Guo X-S, He B, Sun L-J, Peng Y, Dong S-S, et al. Supporting data for " Genome data from sweet and grain sorghum (Sorghum bicolor)". GigaScience Database. 2017. http://dx.doi.org/XXXX

Prior to publication, we will update the citation with the actual doi of the dataset.

Response:
We have added the reference to the dataset as an in-text citation [31] and to the reference list as: Mitterboeck TF, Liu S, Adamowicz SJ, Fu J, Zhang R, Song W, et al. Supporting data for "Positive and relaxed selection associated with flight evolution and loss in insect transcriptomes". GigaScience Database 2017. http://dx.doi.org/XXXX
We have replaced all mention of Additional File 1 (AF1) in the manuscript text with the citation [31] to this data set.

Once you have made the necessary corrections, please submit a revised manuscript online at:

http://giga.edmgr.com/

If you have forgotten your username or password please use the "Send Login Details" link to get your login information. For security reasons, your password will be reset.

Please include a point-by-point within the 'Response to Reviewers' box in the submission system. Please ensure you describe additional experiments that were carried out and include a detailed rebuttal of any criticisms or requested revisions that you disagreed with. Please also ensure that your revised manuscript conforms to the journal style, which can be found in the Instructions for Authors on the journal homepage.

The due date for submitting the revised version of your article is 10 Aug 2017.

We look forward to receiving your revised manuscript soon.

Best wishes,

Hans Zauner
GigaScience
www.gigasciencejournal.com

Reviewer reports:
Reviewer #1: This is a fairly concisely and effectively written report of a large comparative genomics study. The hypothesis is that the evolutionary transition to flight, and subsequent

independent losses of flight, in pterygote insects involved detectable changes in selective pressures and adaptation in a large set of single-copy, orthologous nuclear and mitochondrial genes. The results are not as clear-cut as similar studies have shown for birds and especially bats, but the authors do a nice job discussing caveats, particularly the much longer evolutionary time involved in insect divergences, which serves to obscure the signal. Figure 2 contains what I think is the most noteworthy result, and which is very well discussed in L 265-279.

General remarks:
The methods are at the end, so it would be helpful to prepare the reader in the introductory parts regarding the distinction between positive selection and relaxation of purifying selection. Both are detected by an increase in dN/dS, but positive selection requires the ratio to exceed 1.0 (correct? If not, explain). Some introductory discussion of the difference between positive and relaxed selection would enrich the paper and better engage the reader in the results.

Response:
We have added to the beginning of the first results section, at line 156:

"Tests of positive selection were performed for each lineage of interest via branch-site models, which estimate dN/dS ratios at each codon site and between branches such that positive selection is detected in the lineage of interest if a subset of codon sites have dN/dS ratios greater than 1, while the other lineages have ratios less than 1 or equal to 1, indicating purifying selection or neutral evolution, respectively."

We have also added at the beginning of the relaxed selection results, at line 217:

"Postulated relaxed selection was detected by increased dN/dS ratios across the fully-flightless vs. flight-capable branches of the tree (i.e. pooling branches by flight state) calculated for the entire length of each gene tested, as opposed to positive selection which was detected using branch-site models (accounting for dN/dS ratios at each site) on individual lineages of interest."

This sets up how the analyses were conducted and the main differences between positive and relaxed selection analysis, which are 1) dN/dS at sites vs. whole gene length; and 2) individual lineage tested vs. multiple lineages tested together.

We have also added some detail on what the results for functional tests represent in the results section, to allow greater understanding for the reader. In the Table 1 caption, at line 185, we have added:

"Statistical over-representation is tested by modified Fisher's exact tests in DAVID and binomial statistics in PANTHER, with raw p values provided here."

There is only one sentence in this paper (L 390) explaining that changes in gene regulation are important for adaptation. The next sentence mentions the need for future studies of this nature to include non-coding regions, but places this in the context of gene gains and losses. I think there needs to be a clear and unequivocal statement that this study examines only coding regions and

does not consider changes in gene regulatory regions that affect things like gene co-regulation, which are demonstrably important in e.g. energy metabolism. Doing this will help set up the finding about genes involved in the regulation of alternative splicing, which may be the tip of the icebeaker for broader regulatory changes.

Response: We have added the following sentences after the discussion of splicing genes, at Line 406:

"This study examined only coding regions and was not able to consider changes in gene regulatory regions, which affect co-regulation. Co-regulation is important in processes including energy production [53]. Our results that suggest alternative splicing is an important gene functional category for flight evolution may be a symptom of the involvement of regulatory changes in general, which we were not able to test here."

53. Waveren C Van, Moraes CT. Transcriptional co-expression and co-regulation of genes coding for components of the oxidative phosphorylation system. BMC Genomics. 2008;9.

It might be worth discussing how/why a gene would show signals of positive selection with flight loss. That would mean that in multiple loss-of-flight lineages, the same ortholog adaptively diverged from flight-adapted forms. This would require, I think, that specific amino acids within proteins are required for flight. Discussing this in a bit of detail would help both the authors and readers see why this is a rather exotic hypothesis, especially after hundreds of millions of years of flight cabability (and hence no clear path back the way they came). Flight requires high capacity: lots of mitochondria packed with membranes and hugely more oxygen supply than required during rest. Metabolic flux and oxygen conductivity matter enormously. Are these things in any system known to be controlled by particular amino acid substitutions in particular genes? I can't readily think of any examples. I do know of a paper that shows that one alternative splice form of one metabolic gene is required for high metabolic flux in flight muscles: Mol. Biol. Cell 8, 1665-1675.

Response: We have added the suggestion about how a gene would show signal of positive selection, at line 426:

"For a gene to show signals of positive selection in multiple flight loss examples, the same ortholog must have adaptively diverged from flight-capable lineages. Given the long evolutionary history in the flight-adapted state before flight losses occurred and the seeming ease in which flight ability can be 'turned off' developmentally by loss of function of specific genes [54], the relaxed selection tests may be more able to uncover trends in genes associated with flight loss than positive selection. Even so, given the consistent association between flight loss and increased reproductive ability, future studies using more genomic information may uncover positive selection with flight loss that we did not, or were not able to, detect here."

We thank the reviewer for the insightful suggestion. We could not think of any further biological interpretation than previously given (lines 422-426, reproduction, sugar metabolism) for the expected functional categories associated with the positive selection analysis of flight loss, and

we did not want to risk over-interpreting our results.

The underlying idea in this study is that flight capability is the major selective factor for flighted vs. unflighted insects. I don't dispute that, but it is worth mentioning that a flight-to-flightless transition will likely involve major changes in ecology and life history, which could include diet, predators, dispersal, phenology, courtship, and so forth. These factors are likely to be species-specific. How would this affect the present results? (I noticed belatedly that the paper discusses this briefly in terms of expecting positive selection in loss of flight lineages arising from dispersal-reproduction tradeoffs. I think you could do more in this regard.)

Response: We have added at line 412:
"The loss of flight is not only associated with the change in flight ability, but also major changes in ecology and life history, such as diet, predation, habitat (e.g. woodlands, deserts), courtship, and often reduction in dispersal ability [8][17]. Such changes are specific to certain species or clades, and thus the use of multiple lineages may help to eliminate some noise created by confounding biological or ecological factors. Nonetheless, some associated factors, such as reduced dispersal ability, are likely commonly associated with flight loss, and therefore, the results here are likely impacted by co-occurring factors in addition to change in flight capability itself."

Specific remarks:
L73. 406 MYA grossly overstates the precision of what we know about this date. You should say something like "approximately 400 MYA".

Response: 406 has been changed to 400 in this sentence (line 73) and also at line 310, both of which now read "approximately 400 million years ago".

L.75. There is a more recent paper that is the more appropriate citation because it addresses more basal insects: PNAS 112 (52), 15946-15951 (2015).

Response: Thank you for the suggestion. We have added this citation (line 75). We opted to keep the other citations as well to show the diversity of hypotheses on insect flight origin.

L78. "at least incomplete metamorphosis". This is awkward wording and difficult to understand until reaching the later part of the sentence. Rewrite this sentence.

Response: This sentence has been reworded as (line 77):
"In addition to the evolution of flight, pterygote insects evolved incomplete metamorphosis, which involves egg, nymph, and adult stages."

Based on this change we also added "within Pterygota" in the following sentence (line 79) to clarify that other metamorphosis types occur in pterygotes in addition to incomplete metamorphosis.

L100-106. Here it you should also state that the the particular splice form of certain genes has been shown to be necessary for insect flight (e.g. Mol. Biol. Cell 8, 1665-1675) and that the relative abundance of different splice variants affects the power output of flight muscle in a basal pterygote (Journal of Experimental Biology 204 (20), 3457-3470). Doing so may help explain the result, although without any genes being named or shown in a table (consider doing so, it's only 39 genes) I can't tell if any of these are known to be related to anything related to metabolism, flight, or life history.

Response: we have added this information at line 112, along with the two references:

“Additionally, particular splice forms of certain genes such as encoding glycerol-3-phosphate dehydrogenase (functions in the glycolytic pathway to produce ATP) appear necessary for flight [27], with the relative abundance of various splice variants affecting the power output of flight muscles, as shown in a dragonfly species [28].”

27. Wojtas K, Slepecky N, Laurence VK, Sullivan D. Flight muscle function in Drosophila requires colocalization of glycolytic enzymes. Mol Biol Cell. 1997;8:1665–75.

28. Marden JH, Fitzhugh GH, Girgenrath M, Wolf MR, Girgenrath S. Alternative splicing, muscle contraction and intraspecific variation: associations between troponin T transcripts, Ca2+ sensitivity and the force and power output of dragonfly flight muscles during oscillatory contraction. J Exp Biol. 2001;204:3457–70.

We have added some candidate gene names to the text in each results section, where the genes relate to wing or mitochondrial functions. Often, genes do not have descriptive names, so we opted to provide some examples in the main prose, rather than the whole candidate gene lists, which would be long given the multiple tests.

Additionally, we have now provided the list of all gene names and descriptions in the Supplementary Material Table S15, with information columns to be able to sort the genes by analysis (e.g. candidate, background, analysis 1, 2, 3). As well, we have added the DAVID gene ontology category results in the Supplementary Material Table S16, in case of database changes.

In conjunction with the other reviewer’s suggestion to add more detailed gene category information, we have created some categories of genes of potential interest by grouping together similar GO terms, 1) related to wing development, and 2) related to mitochondrion, ATP-binding, and the electron transport chain, etc. Grouping of GO terms is suggested in the literature to improve detection of trends since multiple terms overlap in their gene lists. The individual GO categories of interest do not show up the candidate gene lists from the DAVID analysis, unless they are over-represented. This way, we have performed the statistical tests ourselves and reported the results for those particular categories of interest. However, there were no significant over- or under-representation of these gene categories in the candidate gene lists, and so the interpretation was not affected by these additions.

The new sections with the gene names and results for grouped functional terms are at lines 165-

174 (analysis 1), lines 199-203 (analysis 2), and lines 226-230 (analysis 3).

L 112-113: "... during the time when flight originated, during a time span of approximately 14 million years [2]." This is similar to my remark about L73 (above). 14MYA is an estimate from a molecular evolution study, and there is very scant (arguably no) fossil evidence for the time of origin of pterygotes. What do you think the error bounds are in this 14 MYA number? I'd guess at least 40 MY and possibly much more.

Response: We have removed the phrase “during a time span of approximately 14 million years” from line 123. We have also removed a similar time estimate from molecular study from line 532 “each representing a time span of an estimated 20 million years”.

342 " ... energy levels needed for multiple flight performances". Not clear what this means. I think you mean to say "allow quantitative adjustment of muscle force and power output". Another meaningful reference here is: Journal of Experimental Biology 211 (23), 3653-3660.

Response: We have changed the phrasing to the reviewer’s suggested phrasing and have added the suggested reference to the sentence, at line 393:
“Alternative splicing allows various isoforms of muscle-related molecules and as such appears to be an important mechanism to allow quantitative adjustment of muscle force and power output [50][51]”

51. Marden JH, Fescemyer HW, Saastamoinen M, Macfarland SP, Vera JC, Frilander MJ, et al. Weight and nutrition affect pre-mRNA splicing of a muscle gene associated with performance, energetics and life history. J Exp Biol. 2008;211:3653–60.

L. 348: I don't understand this sentence. What does "flight morphs within species" mean in the context of this study? How does this sentence relate to the next sentence?

Response: the two sentences have been rephrased for clarity, at line 401-404, as:

“However, flightless vs. flying morphs of insects do not represent evolutionarily distinct lineages, and so genes exhibiting different expression levels among morphs may not be those bearing signatures of differences in selection regime between flightless vs. flying insects on much longer evolutionary timescales. Thus, we suggest genes related to splicing are a potential category for further investigation of whether differing selection pressures occurred with the origination of flight and flight loss in insects.”

Reviewer #2: The authors use data from the 1KITE project and additional genomic data from Misof et al. (2014, Science) to infer positive/relaxed selection on a selection of over 1000 nuclear and mitochondrial genes that are potentially involved in the loss/gain of insect flight.

Overall, I thought the manuscript was well written and the analyses very thorough albeit with limited conclusions. The authors use a comprehensive sequence data set from diverse insect fauna and try to match the phylogeny of flight gain/loss with models of dN/dS as measures of selection. In my opinion the main conclusions are (i) the evidence for relaxed selection in mtOXPHOS genes mirroring that of other taxa and (ii) the association of 'splicing' with flight loss although it would have been nice to see some extension work to the latter finding rather than simple speculation in the Discussion.

As I say I don't think the results are ground-breaking but I did appreciate the authors' efforts to spell out the caveats with their approach and in doing so they mainly addressed my comments as I was reading the paper. For example, I would be keen to see the data for more specialised gene categories.

Response: We have added analyses examining the proportion of genes in the candidate and non-candidate gene lists that fit into potentially relevant gene categories. We created categories of genes of potential interest by grouping together similar GO terms, 1) related to wing development, and 2) related to mitochondrion, ATP-binding, and the electron transport chain, etc. Grouping of GO terms is suggested in the literature, as it can increase the ability to detect trends when GO terms are overlapping. The individual GO categories of interest do not show up the candidate gene lists from the DAVID analysis, unless they are over-represented. We report the results of these larger categories in the manuscript; however, since the genes in the candidate gene lists do not significant over- or under-represent these categories, the interpretation is not changed.

Additionally, we have now provided the list of all gene names and descriptions in the Supplementary Material Table S15, with information columns to be able to sort the genes by analysis (e.g. candidate, background, analysis 1, 2, 3). As well, we have added the DAVID gene ontology category results in the Supplementary Material Table S16, in case of database changes.

Furthermore (in response to a further comment below), we have added the gene names and their joint functional category for the two genes which were overlapping between the flight gain (positive selection) and flight loss (relaxed selection) analyses, as those two genes were the most likely to be of importance (at lines 253-260).

While the other reviewer suggested adding gene lists into the manuscript, many of the genes do not have descriptive names. So, in the main prose, we opted to report only some examples of genes in the candidate lists, where they fall into these categories of interest.

The new sections with the gene names and results for grouped functional terms are at lines 165-174 (analysis 1), lines 199-203 (analysis 2), and lines 226-230 (analysis 3).

I was also curious as to how you determine whether the signatures of selection are truly related to flight and not some pleiotropic phenotype (e.g. delayed reproduction). But again, these concerns were at least highlighted in the Discussion.

Response: the other reviewer had also suggested more discussion on these potential confounding

factors and so we elaborated slightly on this point, at line 412-419:

"The loss of flight is not only associated with the change in flight ability, but also major changes in ecology and life history, such as diet, predation, habitat (e.g. woodlands, deserts), courtship, and often reduction in dispersal ability [8][17]. Such changes are specific to certain species or clades, and thus the use of multiple lineages may help to eliminate some noise created by confounding biological or ecological factors. Nonetheless, some associated factors, such as reduced dispersal ability, are likely commonly associated with flight loss, and therefore, the results here are likely impacted by co-occurring factors in addition to change in flight capability itself."

Major comments
I see the benefits of the GO-term approach but is this a bit simplistic? Especially when you are only sampling from a relatively small number of genes (n = ~1000). I was wondering whether there is any way to correlate dN/dS with the timing of flight gain/loss from your phylogenetic records - would any correlation in say, one of the genes showing relaxed selection associated with flight loss, provide useful additional information?

Response: We thank the reviewer for this interesting idea. We understood the suggestion to mean to consider testing whether more ancient cases of flight loss show higher dN/dS ratios than more recent cases of flight loss for particular genes, and that these genes may be of interest. We have considered the suggestion, but did not think it feasible with the current state of the data. Based on the reviewers' other suggestions about the imprecision of timing events with molecular phylogenies, as well as our other mentioned caveat about the sampling of lineages making it difficult to pinpoint the origin of some cases of these flight loss on the phylogeny, we were not confident that those results would prove accurate. This is certainly an approach to consider for future work involving trees with higher sampling of lineages that would have more accurate divergence times between flightless lineages and closest flight-capable sister lineages.

However, we did provide more specific information at the level of genes, as opposed to only examining overlap in the broader GO categories. We have now reported which genes overlap between those detected under positive selection with flight gain and those detected under relaxed selection with flight loss, as we expect those genes showing mirror-image similarities between the two transition directions to be the most relevant (at lines 253-260).

Minor comments:
Line 57 – proteases

Response: This has been changed to "proteases" (line 57)

Line 71 - 'likely positively' is not correct terminology

Response: This phrase has been changed to "most likely had a positive impact on" (line 71)

Line 82 - 'an estimated thousand of times' doesn't sound right

Response: we have changed this phrasing to "it has been estimated that flight has been lost thousands of times within pterygotes" (line 82-83)

Background, paragraph 3 - the authors may be interested in differentially expressed candidate gene sets associated with flight activity in the Glanville fritillary butterfly (Kvist et al., 2015, Molecular Ecology) and the cotton bollworm moth, Helicoverpa armigera (Jones et al. 2015, Molecular Ecology) to enhance discussion and comparisons.

Response: We have added one sentence with information from both references at line 108-112:

"Functions of genes observed to be differentially expressed between flying insect individuals with higher vs. lower flight metabolic rate include ribosome/RNA processing [25], while genes exhibiting differences between long- vs. short-distance flight migrators include those involved in lipid mobilization and flight muscle structure [26]."

25. Kvist J, Mattila ALK, Somervuo P, Ahola V, Koskinen P, Paulin L, et al. Flight-induced changes in gene expression in the Glanville fritillary butterfly. Mol Ecol. 2015;24:4886–900.

26. Jones CM, Papanicolaou A, Mironidis GK, Vontas J, Yang Y, Lim KS. Genomewide transcriptional signatures of migratory flight activity in a globally invasive insect pest. Mol Ecol. 2015;24:4901–11.

We have received recommendations for additional references and information from the other reviewer as well, so we chose to keep each addition relatively short, in line with the previous concise descriptions earlier in the paragraph of the introduction.

Line 134 – guidelines

Response: "guideline" has been changed to "guidelines" (line 144)

Line 198 - were significant

Response: We have changed "four of those as significant difference" to "four of those exhibited a significant difference" (line 234)

Line 199 - what does ~ mean in the context of '~0'?

Response: We have replaced "~0" with "1.0x10-16" (line 235)

I think for the lay reader at times in the main body of text it would be nice to include an example of a common name species related the insect Order (e.g. Thysanoptera, thrips). It helps the reader and improves the accessibility of the paper.

Response: We have added common names within lines:

Line 63 (abstract): “holometabolous (complete metamorphosis)”
Line 269-271: “The apterygote lineages (i.e. primarily flightless lineages, highlighted in grey) as well as lineages in orders Odonata (i.e. dragonflies and damselflies) and Ephemeroptera (i.e. mayflies),”
Line 277: “holometabolous (i.e. complete metamorphosis) insect clade”
Line 350-351: “Hymenoptera (i.e. bees, wasps, ants, sawflies), Coleoptera (i.e. beetles), Strepsiptera (i.e. twisted-wing parasites), Diptera (i.e. flies), and Thysanoptera (i.e. thrips) [41].”

Other changes:
We have modified a previous phrase in the acknowledgment section to add our thanks to the two reviewers by the names provided in their reviews, at line 677-678:

“and Jim Marden and Christopher Jones for valuable input on the manuscript as reviewers.”

In results section 1 (line 174-175) we changed the Fisher’s exact test to be for 39 candidate genes that were unique to the Pterygota lineage instead of 126 candidate genes that were detected but not necessarily unique. The change was made in order to fit with the reporting of newly added results for the 39 unique candidate genes.

As an addition in the discussion caveat section, we acknowledge that our sample size of genes is low within some Gene Ontology categories (line 463-465):
“Many gene functional categories are poorly represented in our data set, and thus the “expected” counts are low in some categories. The results of this study might be therefore be considered hypotheses for testing using a larger portion of genome in future studies.”

We thank the Editor for the careful review of our paper. We think that addressing these comments has strengthened the quality and readability of the paper for future readers.
