# Supplementary material for: Positive and relaxed selection associated with flight evolution and loss in insect transcriptomes
Source: Gigascience. 2017 Aug 16;6(10):1–14. doi: 10.1093/gigascience/gix073 (PMC5632299; doi:10.1093/gigascience/gix073)
Supplement: Reviewer-1-Report-(Original-Submission).pdf [file gix073_Reviewer-1-Report-(Original-Submission).pdf]

Reviewer Report

Title: Positive and relaxed selection associated with flight evolution and loss in insect transcriptomes

Version: Original Submission **Date:** 4/30/2017

Reviewer name: Jim Marden

Reviewer Comments to Author:

This is a fairly concisely and effectively written report of a large comparative genomics study. The hypothesis is that the evolutionary transition to flight, and subsequent independent losses of flight, in pterygote insects involved detectable changes in selective pressures and adaptation in a large set of single-copy, orthologous nuclear and mitochondrial genes. The results are not as clear-cut as similar studies have shown for birds and especially bats, but the authors do a nice job discussing caveats, particularly the much longer evolutionary time involved in insect divergences, which serves to obscure the signal. Figure 2 contains what I think is the most noteworthy result, and which is very well discussed in L 265-279.

General remarks:
The methods are at the end, so it would be helpful to prepare the reader in the introductory parts regarding the distinction between positive selection and relaxation of purifying selection. Both are detected by an increase in dN/dS, but positive selection requires the ratio to exceed 1.0 (correct? If not, explain). Some introductory discussion of the difference between positive and relaxed selection would enrich the paper and better engage the reader in the results.

There is only one sentence in this paper (L 390) explaining that changes in gene regulation are important for adaptation. The next sentence mentions the need for future studies of this nature to include non-coding regions, but places this in the context of gene gains and losses. I think there needs to be a clear and unequivocal statement that this study examines only coding regions and does not consider changes in gene regulatory regions that affect things like gene co-regulation, which are demonstrably important in e.g. energy metabolism. Doing this will help set up the finding about genes involved in the regulation of alternative splicing, which may be the tip of the icebeaker for broader regulatory changes.

It might be worth discussing how/why a gene would show signals of positive selection with flight loss. That would mean that in multiple loss-of-flight lineages, the same ortholog adaptively diverged from flight-adapted forms. This would require, I think, that specific amino acids within proteins are required for flight. Discussing this in a bit of detail would help both the authors and readers see why this is a rather exotic hypothesis, especially after hundreds of millions of years of flight cabability (and hence no clear path back the way they came). Flight requires high capacity: lots of mitochondria packed with membranes and hugely more oxygen supply than required during rest. Metabolic flux and oxygen conductivity matter enormously. Are these things in any system known to be controlled by particular amino acid substitutions in particular genes? I can't readily think of any examples. I do know of a paper

that shows that one alternative splice form of one metabolic gene is required for high metabolic flux in flight muscles: Mol. Biol. Cell 8, 1665-1675.

The underlying idea in this study is that flight capability is the major selective factor for flighted vs. unflighted insects. I don't dispute that, but it is worth mentioning that a flight-to-flightless transition will likely involve major changes in ecology and life history, which could include diet, predators, dispersal, phenology, courtship, and so forth. These factors are likely to be species-specific. How would this affect the present results? (I noticed belatedly that the paper discusses this briefly in terms of expecting positive selection in loss of flight lineages arising from dispersal-reproduction tradeoffs. I think you could do more in this regard.)

Specific remarks:
L73. 406 MYA grossly overstates the precision of what we know about this date. You should say something like "approximately 400 MYA".

L.75. There is a more recent paper that is the more appropriate citation because it addresses more basal insects: PNAS 112 (52), 15946-15951 (2015).

L78. "at least incomplete metamorphosis". This is awkward wording and difficult to understand until reaching the later part of the sentence. Rewrite this sentence.

L100-106. Here it you should also state that the the particular splice form of certain genes has been shown to be necessary for insect flight (e.g. Mol. Biol. Cell 8, 1665-1675) and that the relative abundance of different splice variants affects the power output of flight muscle in a basal pterygote (Journal of Experimental Biology 204 (20), 3457-3470). Doing so may help explain the result, although without any genes being named or shown in a table (consider doing so, it's only 39 genes) I can't tell if any of these are known to be related to anything related to metabolism, flight, or life history.

L 112-113: "... during the time when flight originated, during a time span of approximately 14 million years [2]." This is similar to my remark about L73 (above). 14MYA is an estimate from a molecular evolution study, and there is very scant (arguably no) fossil evidence for the time of origin of pterygotes. What do you think the error bounds are in this 14 MYA number? I'd guess at least 40 MY and possibly much more.

342 " ... energy levels needed for multiple flight performances". Not clear what this means. I think you mean to say "allow quantitative adjustment of muscle force and power output". Another meaningful reference here is: Journal of Experimental Biology 211 (23), 3653-3660.

L. 348: I don't understand this sentence. What does "flight morphs within species" mean in the context of this study? How does this sentence relate to the next sentence?

Methods

Are the methods appropriate to the aims of the study, are they well described, and are necessary controls included? Yes

Conclusions

Are the conclusions adequately supported by the data shown? Yes

Reporting Standards

Does the manuscript adhere to the journal's guidelines on [minimum standards of reporting?]() YesChoose an item.

Statistics

Are you able to assess all statistics in the manuscript, including the appropriateness of statistical tests used? Yes, and I have assessed the statistics in my report.

Quality of Written English

Please indicate the quality of language in the manuscript: Acceptable

Declaration of Competing Interests

Please complete a declaration of competing interests, considering the following questions:

- Have you in the past five years received reimbursements, fees, funding, or salary from an organisation that may in any way gain or lose financially from the publication of this manuscript, either now or in the future?
- Do you hold any stocks or shares in an organisation that may in any way gain or lose financially from the publication of this manuscript, either now or in the future?
- Do you hold or are you currently applying for any patents relating to the content of the manuscript?
- Have you received reimbursements, fees, funding, or salary from an organization that holds or has applied for patents relating to the content of the manuscript?
- Do you have any other financial competing interests?
- Do you have any non-financial competing interests in relation to this paper?

If you can answer no to all of the above, write 'I declare that I have no competing interests' below. If your reply is yes to any, please give details below.

I declare that I have no competing interests

I agree to the open peer review policy of the journal. I understand that my name will be included on my report to the authors and, if the manuscript is accepted for publication, my named report including any attachments I upload will be posted on the website along with the authors' responses. I agree for my

report to be made available under an Open Access Creative Commons CC-BY license (http://creativecommons.org/licenses/by/4.0/). I understand that any comments which I do not wish to be included in my named report can be included as confidential comments to the editors, which will not be published.

I agree to the open peer review policy of the journal

To further support our reviewers, we have joined with Publons, where you can gain additional credit to further highlight your hard work (see: https://publons.com/journal/530/gigascience). On publication of this paper, your review will be automatically added to Publons, you can then choose whether or not to claim your Publons credit. I understand this statement.

Yes
