# Supplementary material for: Positive and relaxed selection associated with flight evolution and loss in insect transcriptomes
Source: Gigascience. 2017 Aug 16;6(10):1–14. doi: 10.1093/gigascience/gix073 (PMC5632299; doi:10.1093/gigascience/gix073)
Supplement: Reviewer-2-Report-(Original-Submission).pdf [file gix073_Reviewer-2-Report-(Original-Submission).pdf]

Reviewer Report

Title: Positive and relaxed selection associated with flight evolution and loss in insect transcriptomes

Version: Original Submission **Date:** 5/9/2017

Reviewer name: Christopher Jones

Reviewer Comments to Author:

The authors use data from the 1KITE project and additional genomic data from Misof et al. (2014, Science) to infer positive/relaxed selection on a selection of over 1000 nuclear and mitochondrial genes that are potentially involved in the loss/gain of insect flight.

Overall, I thought the manuscript was well written and the analyses very thorough albeit with limited conclusions. The authors use a comprehensive sequence data set from diverse insect fauna and try to match the phylogeny of flight gain/loss with models of dN/dS as measures of selection. In my opinion the main conclusions are (i) the evidence for relaxed selection in mtOXPHOS genes mirroring that of other taxa and (ii) the association of 'splicing' with flight loss although it would have been nice to see some extension work to the latter finding rather than simple speculation in the Discussion.

As I say I don't think the results are ground-breaking but I did appreciate the authors' efforts to spell out the caveats with their approach and in doing so they mainly addressed my comments as I was reading the paper. For example, I would be keen to see the data for more specialised gene categories. I was also curious as to how you determine whether the signatures of selection are truly related to flight and not some pleiotropic phenotype (e.g. delayed reproduction). But again, these concerns were at least highlighted in the Discussion.

Major comments
I see the benefits of the GO-term approach but is this a bit simplistic? Especially when you are only sampling from a relatively small number of genes (n = ~1000). I was wondering whether there is any way to correlate dN/dS with the timing of flight gain/loss from your phylogenetic records - would any correlation in say, one of the genes showing relaxed selection associated with flight loss, provide useful additional information?

Minor comments:
Line 57 - proteases
Line 71 - 'likely positively' is not correct terminology
Line 82 - 'an estimated thousand of times' doesn't sound right
Background, paragraph 3 - the authors may be interested in differentially expressed candidate gene sets associated with flight activity in the Glanville fritillary butterfly (Kvist et al., 2015, Molecular Ecology) and the cotton bollworm moth, Helicoverpa armigera (Jones et al. 2015, Molecular Ecology) to enhance discussion and comparisons.

Line 134 - guidelines
Line 198 - were significant
Line 199 - what does ~ mean in the context of '~0'?
I think for the lay reader at times in the main body of text it would be nice to include an example of a common name species related the insect Order (e.g. Thysanoptera, thrips). It helps the reader and improves the accessibility of the paper.

Methods

Are the methods appropriate to the aims of the study, are they well described, and are necessary controls included? Yes

Conclusions

Are the conclusions adequately supported by the data shown? Yes

Reporting Standards

Does the manuscript adhere to the journal's guidelines on minimum standards of reporting? YesChoose an item.

Statistics

Are you able to assess all statistics in the manuscript, including the appropriateness of statistical tests used? Yes, and I have assessed the statistics in my report.

Quality of Written English

Please indicate the quality of language in the manuscript: Acceptable

Declaration of Competing Interests

Please complete a declaration of competing interests, considering the following questions:

- Have you in the past five years received reimbursements, fees, funding, or salary from an organisation that may in any way gain or lose financially from the publication of this manuscript, either now or in the future?
- Do you hold any stocks or shares in an organisation that may in any way gain or lose financially from the publication of this manuscript, either now or in the future?
- Do you hold or are you currently applying for any patents relating to the content of the manuscript?
- Have you received reimbursements, fees, funding, or salary from an organization that holds or has applied for patents relating to the content of the manuscript?
- Do you have any other financial competing interests?

- Do you have any non-financial competing interests in relation to this paper?

If you can answer no to all of the above, write 'I declare that I have no competing interests' below. If your reply is yes to any, please give details below.

I declare that I have no competing interests

I agree to the open peer review policy of the journal. I understand that my name will be included on my report to the authors and, if the manuscript is accepted for publication, my named report including any attachments I upload will be posted on the website along with the authors' responses. I agree for my report to be made available under an Open Access Creative Commons CC-BY license (http://creativecommons.org/licenses/by/4.0/). I understand that any comments which I do not wish to be included in my named report can be included as confidential comments to the editors, which will not be published.

I agree to the open peer review policy of the journal

To further support our reviewers, we have joined with Publons, where you can gain additional credit to further highlight your hard work (see: https://publons.com/journal/530/gigascience). On publication of this paper, your review will be automatically added to Publons, you can then choose whether or not to claim your Publons credit. I understand this statement.

Yes
